# Intronic primers reveal unexpectedly high major histocompatibility complex diversity in Antarctic fur seals

**DOI:** 10.1038/s41598-022-21658-7

**Published:** 2022-10-26

**Authors:** Jonas Tebbe, Meinolf Ottensmann, Katja Havenstein, Artemis Efstratiou, Tobias L. Lenz, Barbara A. Caspers, Jaume Forcada, Ralph Tiedemann, Joseph I. Hoffman

**Affiliations:** 1 Department of Animal Behaviour, Bielefeld University, 33501 Bielefeld, Germany; 2 Unit of Evolutionary Biology / Systematic Zoology, Institute of Biochemistry and Biology, University of Potsdam, Potsdam-Golm, Germany; 3 Research Unit for Evolutionary Immunogenomics, Department of Biology, University of Hamburg, Martin-Luther-King-Platz 3, 20146 Hamburg, Germany; 4 Department of Behavioural Ecology, Bielefeld University, 33501 Bielefeld, Germany; 5 British Antarctic Survey, High Cross, Madingley Road, Cambridge, CB3 OET UK

**Keywords:** Population genetics, Immunogenetics, MHC class II, Genetic markers, Evolution, Immunology

## Abstract

The major histocompatibility complex (MHC) is a group of genes comprising one of the most important components of the vertebrate immune system. Consequently, there has been much interest in characterising MHC variation and its relationship with fitness in a variety of species. Due to the exceptional polymorphism of MHC genes, careful PCR primer design is crucial for capturing all of the allelic variation present in a given species. We therefore developed intronic primers to amplify the full-length 267 bp protein-coding sequence of the MHC class II DQB exon 2 in the Antarctic fur seal. We then characterised patterns of MHC variation among mother–offspring pairs from two breeding colonies and detected 19 alleles among 771 clone sequences from 56 individuals. The distribution of alleles within and among individuals was consistent with a single-copy, classical DQB locus showing Mendelian inheritance. Amino acid similarity at the MHC was significantly associated with genome-wide relatedness, but no relationship was found between MHC heterozygosity and genome-wide heterozygosity. Finally, allelic diversity was several times higher than reported by a previous study based on partial exon sequences. This difference appears to be related to allele-specific amplification bias, implying that primer design can strongly impact the inference of MHC diversity.

## Introduction

The major histocompatibility complex (MHC) is among the most important components of the immune system in jawed vertebrates, comprising more than 280 genes and spanning several megabases of the genome in humans^1,2^. Numerous studies have demonstrated the functional importance of MHC genotype, which has been associated with variation in key fitness components such as parasite susceptibility, survival, reproductive success and mate choice across many species^2–6^. Consequently, MHC genes are important candidates for the study of adaptive genetic variation.

Proteins encoded by the classical MHC genes bind foreign peptides derived from parasites and pathogens for presentation to T-lymphocytes and subsequent initiation of an adaptive immune response^7^. The MHC gene family encompasses two main subgroups of classical genes that encode immunologically active molecules with structural and functional similarities^1^. MHC class I and II molecules facilitate the recognition of intracellular parasites such as viruses and extracellular parasites such as bacteria, respectively^7^. Both classes of molecule contain a peptide binding region (PBR) responsible for binding foreign peptides. In the case of MHC class I proteins, the PBR is defined by two α–chain domains, whereas an α– and a β–chain fold together to create the PBR of MHC class II proteins^8^. Both classes of MHC molecules exhibit exceptional genetic variability in most species, which is assumed to be driven by pathogen-mediated balancing selection, with each MHC gene often harbouring tens or hundreds of different allelic variants in a given population^2^.

In order to elicit an immune cascade, binding of an MHC’s PBR to the antigenic peptide is required, whereby a single MHC variant shows specificity to a repertoire of peptides that share common amino acid residues at anchor positions^9^. Consequently, heterozygosity at MHC loci may confer enhanced resistance to parasites and pathogens by increasing the diversity of antigens that can be recognised and presented to T-cells^10^. It is therefore not surprising that the MHC is in general characterised by high levels of diversity and includes some of the most polymorphic genes described in vertebrates^11^. In humans, thousands of alleles have been characterised for the most polymorphic MHC loci, resulting in a nucleotide diversity that exceeds the genome-wide average by two orders of magnitude^12–14^.

This pattern is particularly pronounced at peptide binding domains of the classical MHC genes, such as the second exons of the MHC class II DQB and DRB loci, which encode functionally important β-1 domains of the MHC class II PBR^8^. The maintenance of polymorphism at these loci is thought to be mediated by pathogen-driven selection, which includes a variety of mechanisms from heterozygote advantage^15^ and divergent allele advantage^16,17^ to negative frequency-dependent selection^11,18^. Sexual selection via MHC dependent mate-choice also appears to be an important force maintaining the diversity of these genes^6,19,20^.

Nevertheless, patterns of allelic diversity at MHC genes show remarkable variation across species. For example, Asiatic black bears are highly polymorphic at the DRB, Scandinavian brown bears have relatively high levels of polymorphism at the DQB and polar bears are characterised by comparable diversity at both genes^21–24^. Moreover, several marine mammal species including narwhals^25^, southern and northern elephant seals^26,27^, fin and sei whales^28^ and polar bears^23^ appear to carry very little variation at the DQB and/or DRB. This observation has led some to argue that marine mammals may be exposed to fewer pathogens in the marine environment than their terrestrial counterparts^23,26^. However, several species including humpback whales^29^, California sea lions^30^ and crabeater seals^31^ appear to buck the trend, implying that species-specific factors play an important role in shaping global patterns of MHC diversity.

Comparing the diversity of a given locus across species can also be challenging from a technical standpoint due to the variety of methods that are applied to amplify MHC alleles, ranging from differences in PCR primer design to a multitude of different sequencing approaches that all represent potential sources of bias^32,33^. The importance of primer design is particularly underappreciated, with many investigators using PCR primers developed and applied in previous studies of other (more or less related) species^33^. However, these primers may not always amplify all of the variation present in the species of interest, leading to unreliable genotyping^33^. This can occur for two main reasons. First, the use of primers developed from partial exon sequences will produce truncated sequences that may exclude variable positions within the exon that flank the primer binding sites. Second, genetic variation within the primer binding sites can inadvertently lead to the incomplete detection of the targeted alleles due to PCR amplification biases, with primer-template mismatches reducing the amplification efficiency of certain alleles and ultimately causing allelic diversity to be underestimated^34,35^.

The correct inference of MHC diversity is clearly essential for understanding the selective forces shaping MHC variation within and among species^33,36^. However, there is also a growing appreciation of the need to incorporate the potentially confounding effects of the genomic background into MHC studies. In particular, MHC heterozygosity can potentially be correlated with genome-wide heterozygosity when there is close inbreeding within a population^37^. This might make it challenging to disentangle the effects of homozygosity at the MHC from the loss of fitness caused by the unmasking of deleterious alleles in inbred individuals^38^. Furthermore, selection on MHC genes can also be affected by linked deleterious mutations that can be shielded from selection due to high levels of local heterozygosity^39^. Finally, comparisons of immunogenetic and neutral diversity can provide insights into mechanisms of selection^40^. For example, balancing selection tends to increase within-population diversity at the MHC relative to selectively neutral loci, leading to weaker population structure at the former^41^, while diversifying selection on the MHC can result in the opposite pattern^42^.

Pinnipeds are ideally suited to studying adaptive variation at the MHC. Despite spending much of their lives offshore, many pinniped species breed at high densities in terrestrial colonies, where they can be exposed to a variety of parasites and pathogens due to the close proximity of conspecifics and the accumulation of faecal material. Furthermore, heterozygosity–fitness correlations have been reported for a variety of fitness components in pinnipeds, ranging from parasite resistance^43^ through early survival^44,45^ to mate choice^46^ and reproductive success^47,48^. This suggests that many fitness components are influenced by an individual’s genetic quality, although the exact mechanism(s) remain open to debate^49–51^. Finally, previous studies of pinnipeds have implicated variation at MHC genes in juvenile and adult mortality^52–54^, parasite susceptibility^55^ and reproductive success^53^, implying strong natural (and potentially sexual) selection at the MHC.

The Antarctic fur seal is a highly polygynous pinniped^56^ that breeds seasonally on sub-Antarctic islands, principally South Georgia in the south-west Atlantic^57^. This species exhibits extreme natal site fidelity, with females returning to within a body length of their birth locations to breed^58^, while adults of both sexes are also faithful to breeding sites within and among seasons^59^. A long-term study at Bird Island, South Georgia, has uncovered associations between heterozygosity, quantified from nine microsatellites, and multiple fitness components including survival, body size and reproductive success^45,46,48,60^. While genomic data suggest that these relationships are likely due to inbreeding depression^61,62^, the extent to which MHC genotype may contribute towards fitness variation remains unclear. Arguably, MHC class II genes might be especially important given that bacterial infection is known to be a major cause of mortality in territorial male Antarctic fur seals^63^.

**Figure 1:**
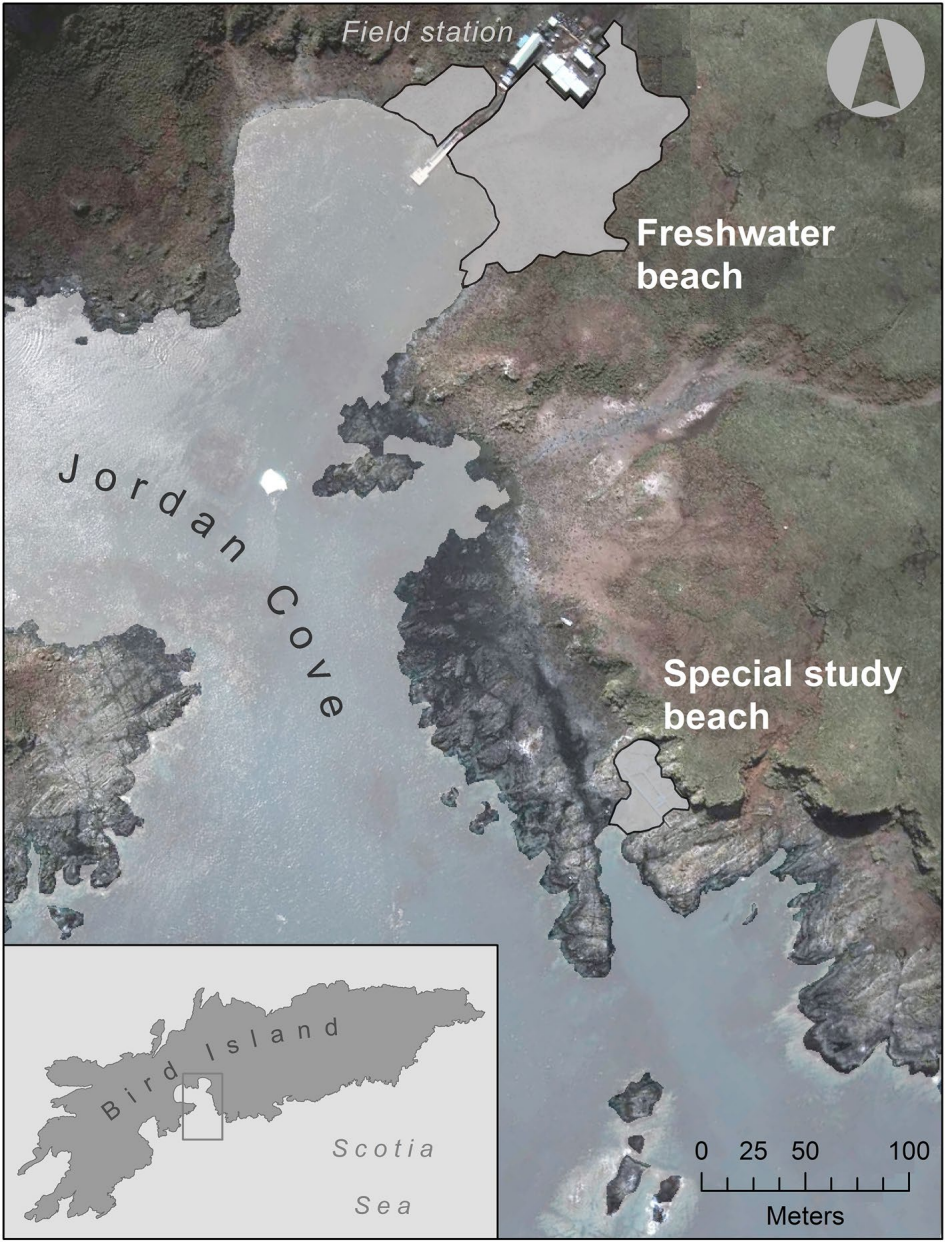
Map of Bird Island, South Georgia, showing the locations of two Antarctic fur seal breeding colonies, the special study beach (SSB) and freshwater beach (FWB). This figure was generated using ArcGIS (version 10.3, ESRI, Redlands, CA, USA, https://www.esri.com/en-us/home) using open source data from Natural Earth Data (https://www.naturalearthdata.com).

Two Antarctic fur seal breeding colonies at Bird Island^64,65^ provide an opportunity to compare and contrast patterns of genetic structure and diversity at the MHC and neutral molecular markers. The Special Study Beach (SSB) and Freshwater Beach (FWB) are situated only around 200 m apart (Fig. 1) yet the density of breeding females is several times higher at SSB (~ 1.2 vs.  ~ 0.3 females per m^2^ respectively)^66^. We have previously shown that pathogenic bacteria are overrepresented in the skin microbial communities of seals from SSB^67^, suggesting that pathogen-driven selection pressures might vary with social density. It is therefore conceivable that selection for specific MHC alleles and/or MHC heterozygosity could be stronger under the more crowded conditions prevailing at SSB.

Here, we designed a novel pair of intronic primers to PCR amplify the full-length protein-coding sequence of the MHC class II DQB exon 2 in Antarctic fur seals. We then used a classical cloning and Sanger sequencing approach to characterise the allelic diversity of mother–offspring pairs from SSB and FWB. The inclusion of close relatives reduced our effective sample size of individuals but allowed us to test for Mendelian mismatches, which is an established approach for identifying genotyping errors^68^. Moreover, the same animals have previously been genotyped at 41 microsatellites^69^, allowing us to compare patterns of diversity at the MHC and putatively neutral genetic markers. We hypothesised that (i) density-dependent selection pressures might lead to contrasting patterns of population structure and/or genetic diversity at the MHC relative to neutral loci; and (ii) a relationship between MHC heterozygosity and genome-wide heterozygosity could potentially arise as a consequence of there being variation in inbreeding within our study population^61,62^. Finally, a previous study by Hoelzel et al.^70^ used PCR primers internal to the DQB exon 2 to amplify partial (141 bp) exon sequences, revealing four alleles among 13 Antarctic fur seal individuals from Bird Island. This set the expectations for our study in terms of MHC diversity, although we anticipated that a combination of a larger sample size of individuals and full-length exon sequences might result in the discovery of some additional alleles.

## Results

Intronic PCR primers were used to amplify a 459 bp product containing the full 267 bp MHC class II protein-coding DQB exon 2 sequence in 44 Antarctic fur seal mother–offspring pairs from two breeding colonies at Bird Island, South Georgia (Fig. 1). PCR products of the expected size were obtained for 56 individuals, comprising 20 mother–offspring pairs plus 12 mothers without sequenced pups and 4 pups without sequenced mothers (see Supplementary Table 1 for details). Cloning and sequencing of these PCR products generated a dataset of 977 full length DQB exon 2 sequences.

### Characterisation of DQB II alleles

**Figure 2:**
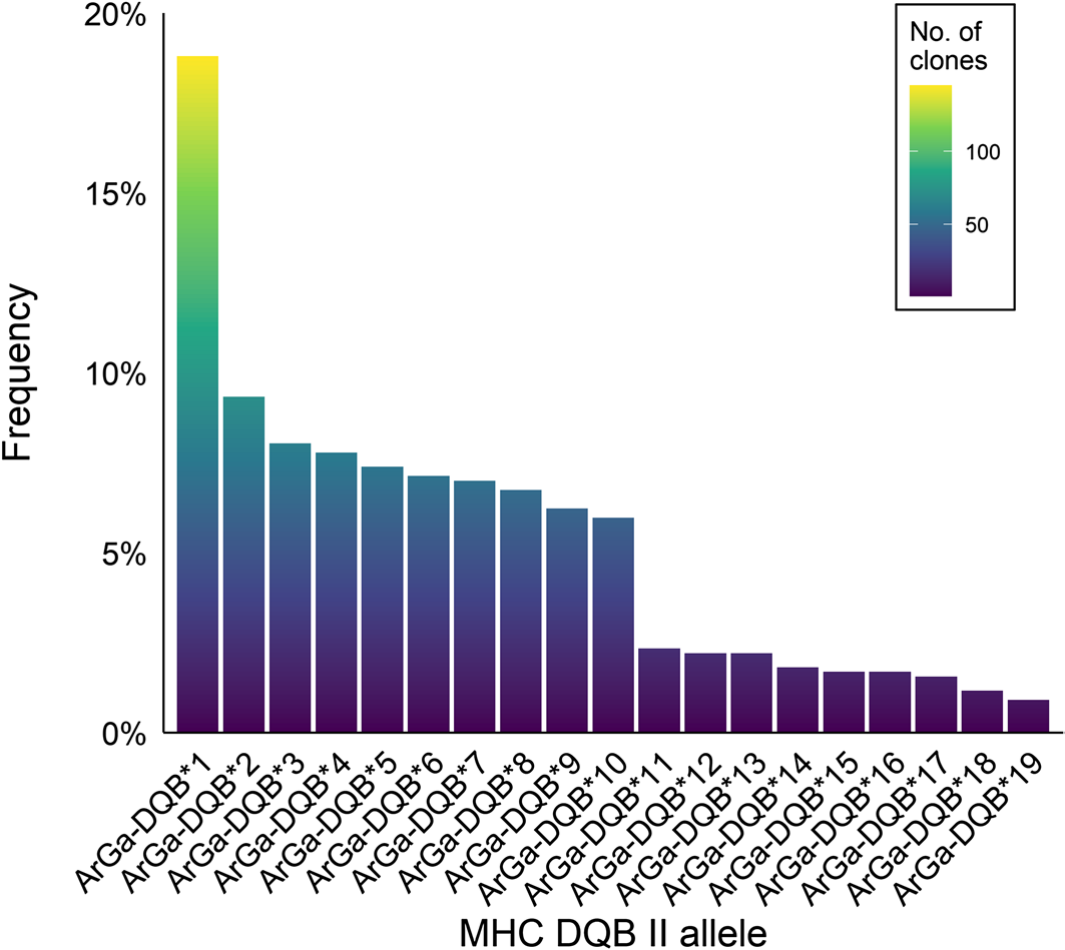
Histogram showing the frequencies of 19 MHC class II DQB exon 2 alleles identified in 56 Antarctic fur seal individuals. Each allele is colour coded according to the total number of clones of that allele in the final cloning dataset, as shown in the accompanying legend.

**Figure 3:**
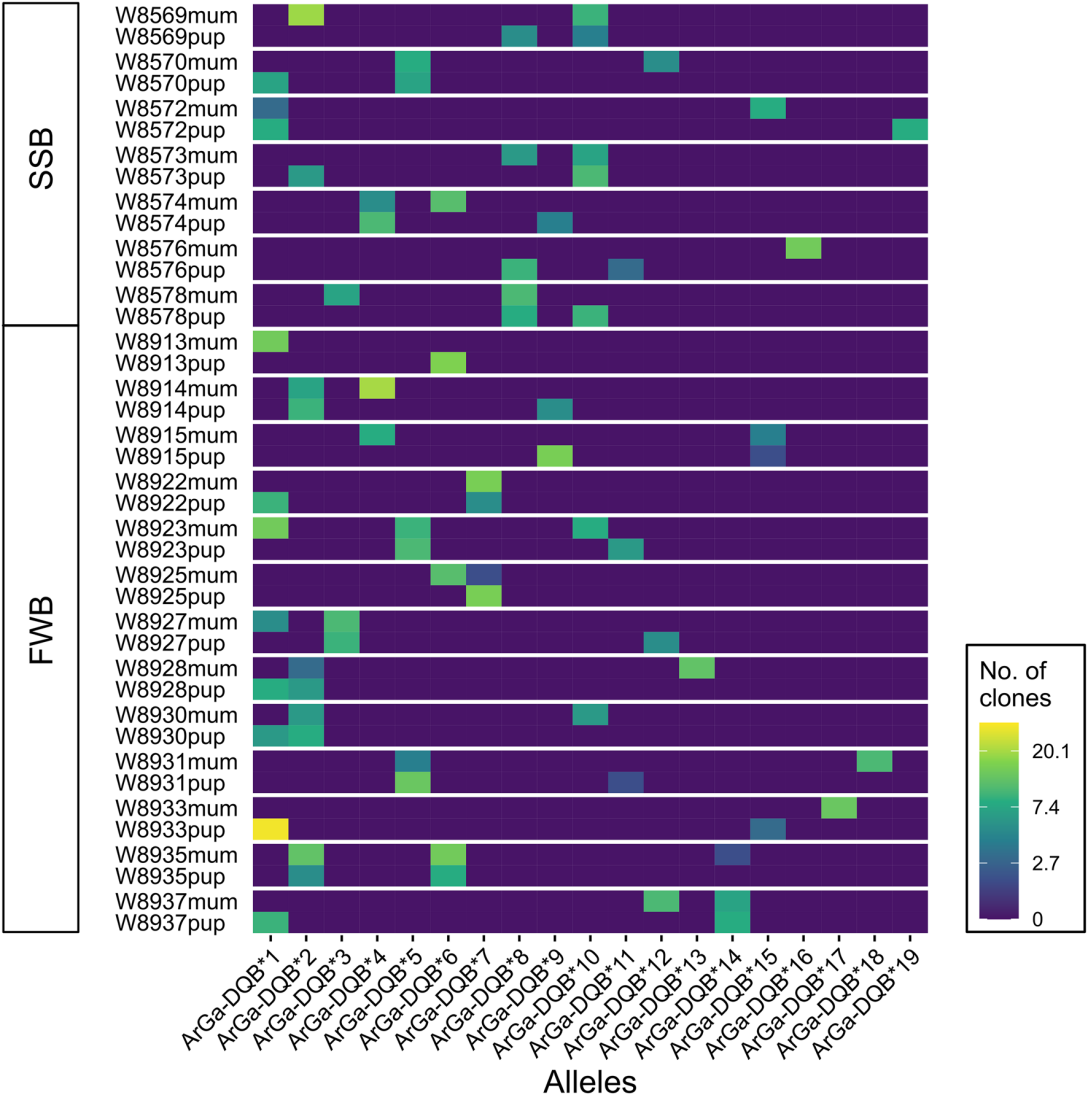
Heatmap depicting the MHC genotypes of 20 Antarctic fur seal mother–offspring pairs from SSB (top) and FWB (bottom). The alleles are colour coded according to the number of clone sequences of that allele obtained for each individual.

184 sequences (9.3%) occurred once in the full dataset (i.e. they were represented by a single clone) and a further ten sequences (1.2%) occurred twice, but always within the same individual. As these sequences differed by one (or at most two) nucleotides from a more common sequence, we classified them as putative artefacts and filtered them out of the dataset. This left a total of 771 clone sequences (average = 13.77, range = 7–32 per individual). Among these sequences, we identified 19 distinct ArGa-DQB alleles, each of which occurred in at least two individuals and was represented by a minimum of seven clone sequences (mean = 40.6; Fig. 2 and Supplementary Table 1). All but two of the animals in our final dataset carried either one or two ArGa-DQB alleles (Supplementary Table 1), consistent with the amplification of a single locus. Furthermore, a comparison of the genotypes of mothers and their pups showed that the majority of pairs (17/20, 85.0%) shared at least one allele (Fig. 3), suggesting that this locus follows Mendelian inheritance.

### Amino acid divergence and patterns of selection

All 19 ArGa-DQB allele sequences translated into unique amino acid sequences, comprising the 89 amino acid residues of the entire exon. Alignments of the amino acid sequences revealed no frameshifts and no premature stop codons. A sequence alignment revealed 34 and 22 variable sites for the nucleotide and amino acid positions respectively, the latter including 13 variable putative Antigen Binding Sites (pABSs; Supplementary Fig. 1). However, it should be noted that pABSs are inferred from sequence homology to the human MHC and it remains unclear whether the direct involvement of these sites in antigen binding is conserved across mammals. At these pABS codons, the three alleles ArGa-DQB*01, ArGa-DQB*06 and ArGa-DQB* 16, and the pair ArGa-DQB08* and ArGa-DQB*13 were identical, respectively. Overall, pairwise amino acid divergence was more than four times higher at the pABS (*d* = 0.304, s.e. = 0.079) than at the non-pABS codons (*d* = 0.066, s.e. = 0.023, *t* = 28.814, *p* < 0.001). Furthermore, non-synonymous substitutions occurred significantly more often than synonymous substitutions at the pABS but not at the non-pABS codons (Table 1), suggesting that the ABS is evolving under balancing selection. In line with this, a maximum likelihood phylogenetic tree (Supplementary Fig. 2) revealed some degree of clustering of ArGa-DQB alleles, but there were also multiple instances of Antarctic fur seal sequences grouping together with alleles from other species including New Zealand fur seals (ArFo), California sea lions (ZaCa) and the walrus (OdRo), indicating potential trans-species polymorphism.

**Table 1:** Non-synonymous (dN) and synonymous (dS) substitution rates at the MHC class II DQB exon 2.

	dN	dS	HA: dN ≠ dS		HA: dN > dS	
			Z	*p*	Z	*p*
Exon	0.41 ± 0.09	0.06 ± 0.03	3.194	0.002	3.077	0.001
pABS	0.78 ± 0.18	0.00 ± 0.00	4.064	< **0.001**	4.154	< **0.001**
Non-pABS	0.23 ± 0.08	0.08 ± 0.04	0.991	0.324	0.985	0.163

*p*-values highlighted in bold indicate significant Z-statistics supporting the tested hypotheses based on modified Nei–Gojobori models with Jukes-Cantor correction. *pABS* putative antigen binding sites.

### Comparisons between colonies and genetic markers

**Figure 4:**
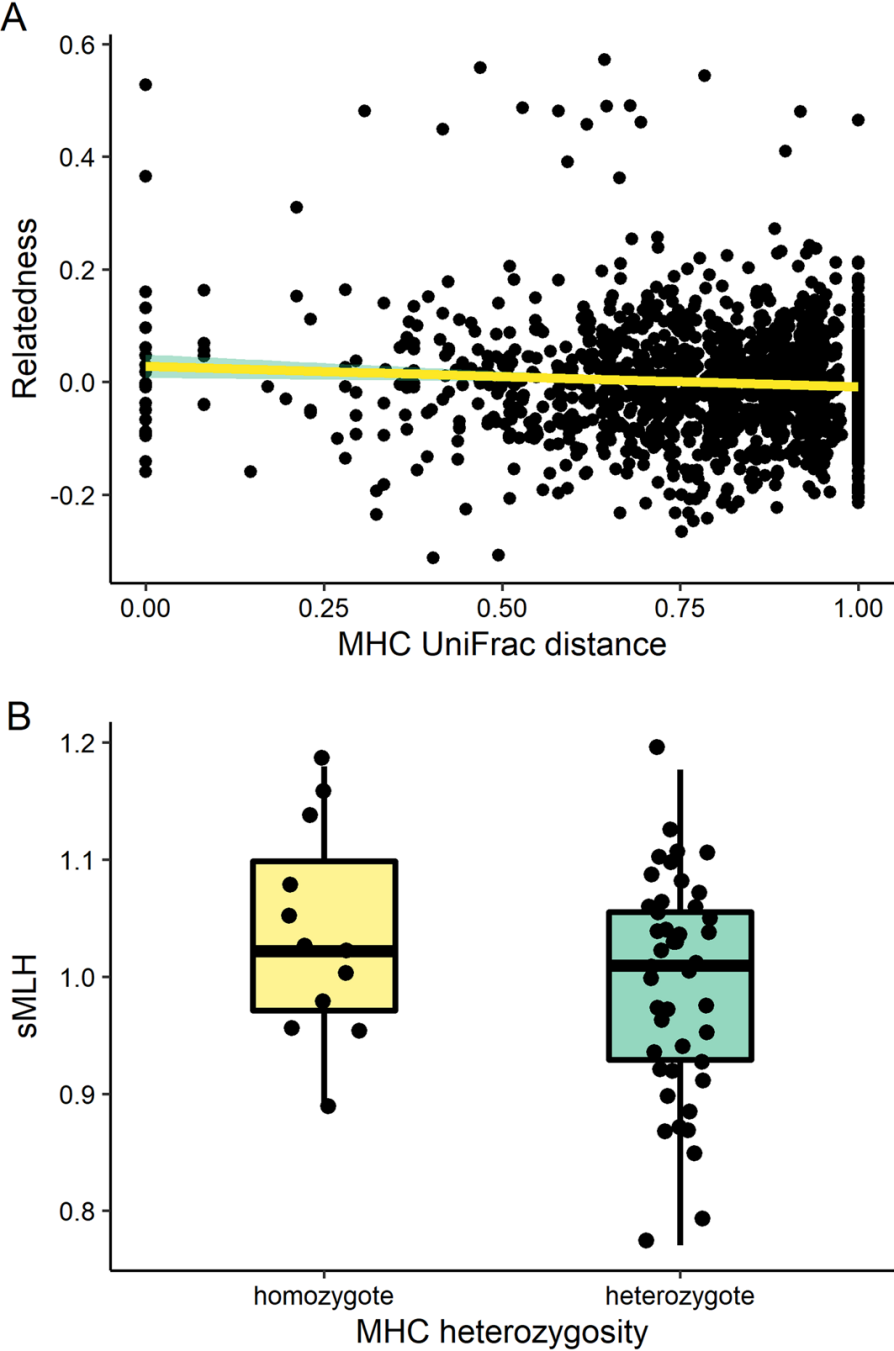
Relationship between (**A**) pairwise MHC UniFrac distance and microsatellite relatedness; (**B**) MHC heterozygosity and microsatellite standardised multilocus heterozygosity.

No significant differences were detected between the two breeding colonies at either the MHC (*F*_st_ = 0.0054, *p* = 0.527) or 41 microsatellites (*F*_st_ = 0.0028, *p* = 0.497). Genetic diversity statistics were also similar for the two colonies (Supplementary Table 2). At the individual level, we detected a weak but statistically significant relationship between pairwise UniFrac distance at the MHC and pairwise microsatellite relatedness (LME, *F*_1,1538_ = 7.908, *p* = 0.005, Fig. 4A). However, MHC heterozygosity was not significantly associated with microsatellite heterozygosity (sMLH, Binomial GLM, χ^2^_1,54_ = 64.50, *p* = 0.155, Fig. 4B).

### Comparison with the previous study

**Figure 5:**
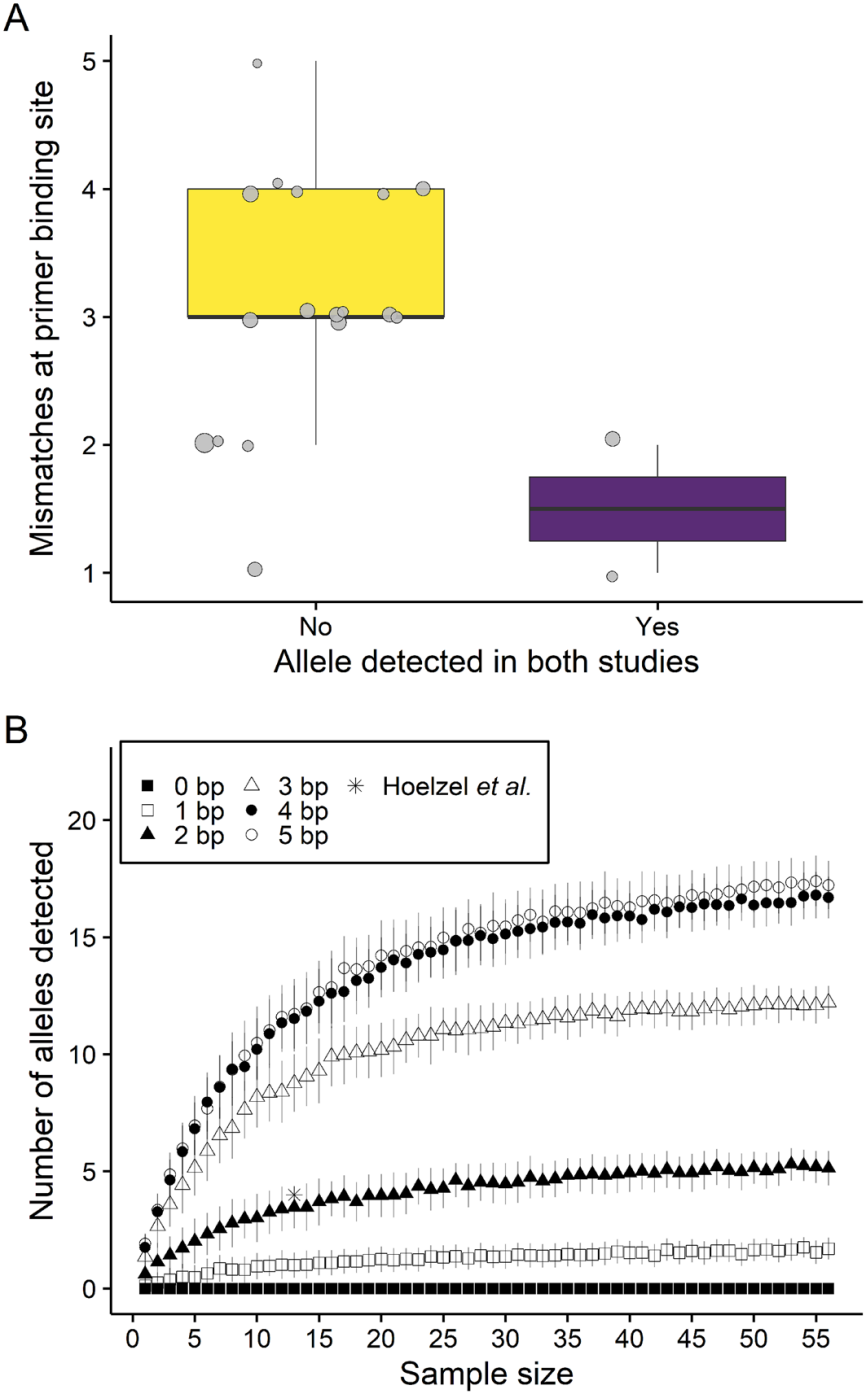
An exploration of possible factors affecting the inference of ArGa-DQB allelic diversity. (**A**) Comparison of the number of nucleotide mismatches at the primer binding sites of Hoelzel et al.^70^. For 17 alleles that uniquely amplified in the current study versus two alleles that were detected in both studies. The box plots show the median and interquartile range, with the whiskers indicating the 95% confidence intervals. Individual alleles are shown with circle size in proportion to allele frequency. (**B**) Allele detection curves computed by randomly sampling the empirical genotypes with replacement (999 replicates each) for sample sizes ranging from one to 56 individuals (see “Materials and methods” section for details). Results are shown separately for different threshold values used to classify alleles based on the number of nucleotide mismatches at the primer binding sites. Shown are the mean and standard deviation of the number of detected alleles.

Hoelzel et al.^70^ used PCR primers internal to the MHC class II DQB exon 2 to amplify four partial (141 bp) exon sequences (therein denoted AFS1–4) in 13 Antarctic fur seal individuals from Bird Island. We detected two of these alleles (AFS1 = ArGa-DQB*17 and AFS2 = ArGa-DRB*06) in our sequence dataset, but AFS3 and AFS4 could not be found among either the alleles or the putative artefacts. To shed light on differences in inferred MHC diversity between the two studies, we compared the number of nucleotide mismatches (Hamming distance) at the primer binding sites used by Hoelzel et al.^70^ for the two alleles that were common to both studies and the 17 alleles found only in the current study. Figure 5A and Supplementary Fig. 3 show that the newly described alleles exhibit on average twice as many nucleotide mismatches at the primer binding sites than the alleles that are common to both studies. This may suggest that sequence variation at the primer binding site could result in biased patterns of amplification among the different alleles. To explore the association between the number of nucleotide mismatches and allelic amplification while accounting for variation in sample size, we computed allele detection curves for our dataset by randomly sampling the genotypes with replacement. Figure 5B shows the relationship between allele number and sample size while setting different threshold values of 1–6 primer binding site mismatches. The total number of discovered alleles increased rapidly with increasing sample size until it approached a plateau at around 30–40 individuals. For a sample size of 13 individuals, we found reasonable concordance between the number of detected alleles showing up to three nucleotide mismatches and the empirical value of Hoelzel et al.^70^, while allowing for additional mismatches resulted in an increasing number of detected alleles.

## Discussion

We developed a novel pair of intronic PCR primers to amplify the full-length protein-coding sequence of the MHC class II DQB exon 2 in Antarctic fur seal mother–offspring pairs from two breeding colonies at Bird Island, South Georgia. We uncovered unexpectedly high levels of MHC diversity, with a total of 19 ArGa-DQB alleles being found in 56 individuals. Allele discovery curves suggest that the discrepancy between our results and those of Hoelzel et al.^70^ cannot be attributed solely to differences in the number of sequenced individuals. Instead, the many alleles that are unique to the current study show more mismatches on average to the primer binding sites used by Hoelzel et al.^70^ than do the few alleles that are common to both studies. One possible explanation for this pattern is that sequence variation at the primer binding sites could have resulted in biased patterns of allelic amplification.

### DQB allele discovery

Historically, attempts at sequencing MHC genes in non-model organisms often relied on PCR primers designed from multiple species alignments of partial exon sequences. By contrast, we were able to use an Antarctic fur seal reference genome to design intronic primers to PCR amplify and sequence the full length MHC class II DQB exon II. This appears to have been instrumental in uncovering a much greater allelic diversity than was expected on the basis of an earlier study^70^. Specifically, our results point towards the presence of at least 19 ArGa-DQB alleles in the South Georgia population. In practice, allelic diversity might be even higher for two reasons. First, we discarded sequences represented by one or two clones, as these mainly differed by one (or at most two) nucleotides from a more common sequence, suggesting that most if not all of them are likely to be PCR or sequencing artefacts, which are common phenomena when PCR products are cloned, as during MHC genotyping^71^. However, this conservative approach may have led to the exclusion of rare genuine alleles that might be underrepresented in our dataset due to chance, stochastic sampling effects. Second, although our sample size of 56 individuals should in principle allow the detection of alleles down to a frequency of around 1%, the true threshold is likely to be higher as many individuals in our dataset are closely related. It is therefore possible that additional alleles might be detected in a larger sample size of unrelated individuals, especially if we could include samples from genetically divergent populations from across the species’ global distribution^62,72^.

The relatively high allelic diversity that we report in the current study is not necessarily unexpected for a polygynous, colonially breeding pinniped where a major cause of adult (but not neonatal) mortality is bacterial infection^63,73^. To investigate whether natural selection could be responsible for maintaining this diversity, it would be necessary to test for associations between MHC genotype and fitness components such as survival, body size and reproductive success, which are already known to be influenced by an individual’s genotype in this species^45,46,48,60^. However, it is also possible that high MHC diversity in Antarctic fur seals could be related to historically large effective population sizes^74^. In support of this hypothesis, a comparative study of ice breeding seals reported very high DQA allelic diversity (39 alleles in 30 individuals) in the world’s most abundant pinniped, the crabeater seal, while diversity was intermediate in Weddell and Ross seals, and lowest in leopard seals^31^. Furthermore, although Antarctic fur seals experienced a strong demographic reduction due to historical sealing, the effective population size during the bottleneck likely remained in the hundreds^72,75,76^. By contrast, the pinniped with the lowest recorded MHC diversity, the northern elephant seal, was hunted down to just a few tens of individuals, which may have resulted in the loss of MHC alleles through genetic drift^27,70^.

In addition to high allelic richness, we also found an excess of non-synonymous substitutions at pABSs, which is suggestive of long-term balancing selection. Such a pattern is not unexpected and is consistent with allelic diversity being maintained over evolutionary time by pathogen-mediated selection. In contrast to the long held view that marine mammals face low pathogen pressures^26^, several studies have described strong effects of MHC diversity on disease susceptibility, survival and reproductive success in pinnipeds^52–55^. Collectively, these studies suggest that natural (and possibly sexual) selection may be an important force in maintaining diversity at the MHC in pinnipeds. Long-term balancing selection and / or adaptation to similar pathogens might also help to explain trans-species polymorphism, which is evident from patterns of allelic similarity among pinniped species. For instance, ArGa-DQB*06 and a New Zealand fur seal allele (AF111044.1) differ by only a single nucleotide and translate into identical protein sequences, while some alleles have been cross identified in further species pairs (e.g. the California sea lion and the Galapagos sea lion [AF503397-398, AF503400-402, AF503406 and HE663128] and the Southern elephant seal and Australian sea lion [AF111038 and KP127614.1]).

### Evidence for a single-copy, Mendelian inherited locus

MHC loci are often duplicated and pinnipeds appear to be no exception. For example, grey seal individuals carry up to four alleles at the DQB exon 2, indicating the presence of at least two loci^77^, while at least seven DRB loci have been identified in California sea lions^30^. By contrast, we found that 54 out of 56 individuals (96.5%) carried one or two ArGa-DQB alleles, pointing towards a single-copy, classical DQB locus. This was further supported by the observation of Mendelian inheritance among the majority of parent–offspring pairs. It is unclear why two individuals (both mothers) each amplified three alleles. One possible explanation could be sample cross-contamination, in which case the genotypes of the individuals in question (see Supplementary Table 1) would be indicative of two independent contamination events. Regardless of the exact explanation, in both cases at least one of the mother’s alleles was shared with her offspring, suggesting that some of the maternal and offspring alleles had been correctly characterised. Another possibility is copy number variation of the MHC class II DQB exon 2. Larger datasets from future studies might shed more light on this aspect.

More generally, we found that the rate of Mendelian mismatches between mothers and offspring was low, with only three pairs out of twenty exhibiting incompatible ArGa-DQB genotypes. We can be confident that these mismatches are not due to mother–offspring pairs having been incorrectly identified in the field^78^ because all of the mothers in the current study matched their pups at 41 microsatellite loci. Instead, all three of the mothers in question appeared to be homozygous for an allele that was not shared with their offspring. The most obvious explanation for this pattern is that these mothers were actually heterozygous for alleles shared with their offspring, but that those alleles went undetected in our cloning dataset due to random allelic dropout or because of PCR amplification bias, which can lead to the preferential amplification of certain alleles. Both have been identified as sources of genotyping error in studies using classical methods capable of screening a limited number of clones^36^.

### Population structure

One approach used to infer selection at the MHC is to compare the strength of population structure at MHC genes and neutral loci. For example, in Scottish grey seals, the DQB exon 2 exhibits stronger differentiation among colonies but a weaker pattern of isolation by distance than microsatellites, which has been interpreted as evidence of habitat-specific selection pressures influencing the genetic composition of breeding colonies^77^. We therefore tested for genetic differences between two Antarctic fur seal colonies that differ in social density. As high social density is often associated with an increased risk of exposure to parasites and pathogens^79^, we hypothesised that animals from these two localities might experience contrasting selection pressures at the MHC. However, we did not find any genetic differences either at the MHC or at 41 microsatellites, with population structure being negligible at both types of marker and genetic diversity being comparable, both overall and when adults were considered separately from offspring. This lack of differentiation at neutral markers suggests that the strong natal philopatry^58^ and adult site fidelity^59^ of this species are not sufficient to generate fine-scale population structure. Furthermore, the absence of any obvious differences in MHC allele frequencies or MHC heterozygosity between animals from the two colonies suggests that the strength of selection on the MHC may not vary with social density. However, our inference is limited in this regard given our modest sample size of individuals together with limited knowledge of density-dependent patterns of pathogen exposure and immune activity in this species.

### Relationship between MHC genotype and the genomic background

Relatively few studies of non-model organisms have combined MHC sequencing with the genotyping of genome-wide distributed, selectively neutral markers such as microsatellites^38^. Consequently, we still have a limited understanding of the relationship between MHC genotype and genome-wide patterns of relatedness and heterozygosity, which is important for understanding mate choice and inbreeding depression. We detected a weak but significant association between genetic relatedness, inferred from microsatellites, and UniFrac distance at the MHC. This reveals the potential for MHC genotype to be involved in the signalling of genetic relatedness in Antarctic fur seals, which could help to explain the ability of females of this species to exert mate choice for partners that are unrelated and heterozygous^46^. However, no clear relationship was observed between MHC heterozygosity and microsatellite heterozygosity, despite the fact that the microsatellites carry a clear signal of identity disequilibrium^69^, indicating that they capture variation in inbreeding within the study population. Although data from many more neutral markers would allow us to be more confident about a lack of association, taken at face value this finding suggests that MHC heterozygosity is not correlated with inbreeding. This could have implications for explaining fitness variation as inbred individuals will not necessarily be more homozygous at the MHC. Consequently, if selection at the MHC contributes to fitness variation in this species, any effects of MHC genotype might occur independently of the loss of fitness that occurs due to inbreeding depression.

### Comparing diversity estimates

Inferred allelic diversity at the MHC class II DQB exon 2 was considerably higher than previously reported for this species^70^. Although we do not have a definitive explanation for this discrepancy, our sequence data clearly show that the primer binding sites of Hoelzel et al.^70^ are variable in Antarctic fur seals from Bird Island. Moreover, our newly described alleles exhibit more mismatches on average at the primer binding sites of Hoelzel et al.^70^ than those alleles that are common to both studies. One explanation for this pattern could be biased allelic amplification, which would appear to be relatively unimportant in the current study due to the use of primers that anneal to the less variable flanking introns. This is consistent with the findings of a metabarcoding study that could explain around 80% of the variation in the amplification success of different arthropod species by the number of mismatches between species-specific priming regions and universal primers^35^. Alternatively, it is unclear exactly where and when the samples analysed by Hoelzel et al.^70^ were collected, although it seems likely they originated from the long-term study colony (SSB) and they must have been gathered prior to the year of publication, i.e. 1999. Thus, it is possible that differences in inferred MHC diversity could be a reflection of the samples having been taken from different time points and locations, although this seems fairly unlikely given that population structure, at least locally, appears rather weak^62,69,72^. Regardless of the exact explanation, our findings lend further support to the argument that primer design is critical for the accurate assessment of allelic diversity at polymorphic genes. Importantly, multiple species alignments are not necessarily informative about intraspecific variation. However, this issue could be circumvented by pre-screening the focal species or population for intraspecific variation at predefined primer binding sites, an approach that is becoming increasingly feasible due to the growing availability of whole genome resequencing data.

### Future perspectives

Although cloning and Sanger sequencing are not amenable to genotyping very large numbers of individuals, the approach we have taken is commonplace at preliminary stages of research and produces a catalogue of known alleles that can be useful for validating results obtained with other methodologies^33^. Our catalogue of ArGa-DQB alleles is somewhat larger than we originally anticipated, but several lines of evidence suggest that most if not all of the characterised alleles are likely to be genuine. Most obviously, they were all cloned from multiple individuals and, with a few exceptions, they appear to conform to Mendelian inheritance. Furthermore, overall patterns of nucleotide divergence and phylogenetic clustering are in line with expected patterns of natural selection and trans-species polymorphism. Future studies should aim to investigate the relationship between MHC genotype and fitness using a larger sample of individuals, ideally while controlling for genome-wide effects, for example using a recently developed 85k SNP array^61^. Another promising research avenue would be to test for the involvement of the MHC in chemical communication^69^ and mate choice^46^.

## Conclusions

We have taken the first steps towards characterizing MHC diversity in Antarctic fur seals. Focusing initially on the MHC class II DQB exon 2, we used intronic primers to identify 19 well-supported alleles. This greater than anticipated diversity appears to be maintained by balancing selection, suggesting that MHC diversity could potentially influence fitness in this species, as is the case for several other pinnipeds^52,53,55,80^. Our results also have methodological implications because they highlight how pre-screening primer binding regions for intraspecific variation could be a worthwhile step in optimizing the discovery of MHC variation in non-model organisms.

## Methods

### Tissue sampling and DNA extraction

Samples were collected from a total of 88 Antarctic fur seal individuals (44 mother–offspring pairs) from two breeding colonies (SSB and FWB) at Bird Island, South Georgia (54° 00′ 24.8″ S, 38° 03′ 04.1″ W). Sampling was conducted in the 2011 breeding season as part of annual routine procedures of the long-term monitoring and survey program of the British Antarctic Survey (BAS). It is unclear when and where the samples analysed by Hoelzel et al.^70^ were collected, although it is likely that these originated from SSB and the sampling must have been conducted prior to 1999. In the current study, skin samples were taken from the interdigital margin of the foreflipper with piglet ear notching pliers and stored at − 20 °C in 20% dimethyl sulphoxide saturated with sodium chloride. DNA was subsequently extracted using a modified chloroform/isoamylalcohol extraction protocol^81^.

### Development of DQB-specific primers

MHC class II DQB exon 2 sequences from multiple pinniped species were extracted from Genbank (See Supplementary Table 3 for details) and aligned within BioEdit^82^. The resulting alignment was then trimmed to 267 bp, corresponding to the entire protein-coding exon 2 sequence, and mapped to the Antarctic fur seal reference genome (version 1.0)^83^ using a BLAST e-value of 1e^-8^ and a word size of 7. This revealed a single match comprising all 267 nucleotides of the protein-coding exon 2 sequence and containing no gaps (Contig 48, 1,937,070–1,937,336). Intronic primers were then designed to amplify the full-length second exon using Primer3Plus^84^ with default settings, except for defining a minimum and maximum melting temperature of 60 °C and 65 °C respectively and limiting the GC content to 35–65%. This identified forward and reverse primers (ArGa-DQB_F: 5′-GCTGTTGGTTGGGCTGAG, ArGa-DQB_R: 5′- CCACCTCAGCAGGAACAGTG) within the conserved flanking regions of the second exon sequence. These primers mapped uniquely to the Antarctic fur seal reference genome using BLAST with an e-value of 10 and a word size of 14 and they were predicted to produce a single product of 459 bp.

### PCR amplification, cloning and sequencing

PCR reactions were performed with 5 μL KAPA HiFi HotStart ReadyMix (KAPA Biosystems, Boston, Unites States), 1 μL of genomic DNA and 2 μL of each primer (1 μM) totalling to a volume of 10 μL. Thermocycling using a TProfessional standard Thermocycler (Biometra GmbH, Göttingen, Germany) comprised an initial denaturation step at 95 °C for 5 min, followed by 30 cycles of 30 s denaturation at 95 °C; 1 min annealing at 70 °C and 1 min extension at 72 °C. A final extension step was performed at 72 °C for 7 min with subsequent cooling to 4 °C. All samples were PCR amplified in triplicate and successful amplification was confirmed by running 1 μL of each PCR product on a standard 2% agarose gel.

The PCR products were purified using a NucleoSpin^®^ Extract II Kit (Macherey–Nagel, Düren, Germany) and incubated with 1 U Taq-polymerase and 0.13 mM dNTPs (both Qbiogene, Heidelberg, Germany). We cloned the amplified MHC fragments into electrocompetent *Escherichia coli* cells (ElectrocompTM TOP10 cells, Invitrogen) using the TOPO TA Cloning^®^ Kit for Sequencing (Invitrogen). Bacterial plasmid inserts were sequenced on an ABI 3100 or ABI 3130 automated sequencer (Applied Biosystems, Foster City, CA, USA), using standard T3 or T7 primers and the BigDye^®^ Terminator v3.1 Cycle Sequencing Kit (Applied Biosystems).

### Sequence analysis

Individual clone nucleotide sequences were aligned using MEGA version 10.1.8^85^. Sequencer trace files for each sequence were manually checked for ambiguous nucleotides and corrected where necessary. All of the nucleotide sequences were then aligned to the MHC class II DQB exon 2 of the Antarctic fur seal reference genome with ClustalW implementation in MEGA (Gap opening penalty 15.00; gap extension penalty 6.66 for both pairwise and multiple alignments) and were subsequently trimmed to a length of 270 bp. We then used the Biostrings package^86^ distributed by the Bioconductor project to build customized functions to handle the sequence data in R.

### Population differentiation and genetic diversity

We generated genetic diversity statistics (the number of private alleles, allelic richness, observed and expected heterozygosities and *F*_is_) for the two colonies using inbreedR^87^, adegenet^88^, vegan^89^ and hierfstat^90^. We tested for genetic differentiation between the two colonies by calculating *F*_st_ separately for the MHC and 41 microsatellites after Weir and Cockerham^91^ within hierfstat. Statistical significance was determined by permuting the original data 9999 times to create a simulated null distribution of *F*_st_ values with ade4^92^. We quantified pairwise MHC UniFrac distance and microsatellite relatedness for all individuals. UniFrac is a distance metric based on the fraction of total unshared branch lengths between pairs based of phylogenetic distances. To perform this analysis, we first built a phylogenetic tree of the MHC class II DQB exon 2 alleles identified in this study using a neighbour-joining model in MEGA^85^ while incorporating *p*-distances to resolve the proportion of nucleotide sites at which two sequences differ. Pairwise genetic relatedness was calculated from the 41 microsatellites as Queller and Goodnight’s *r* using Demerelate^93^. Additionally, we quantified MHC haplotype heterozygosity (1 = heterozygous, 0 = homozygous) as well as standardised multilocus heterozygosity (sMLH) from the microsatellites using inbreedR^87^. To test for an association between pairwise UniFrac distances at the MHC and relatedness, we constructed a linear mixed effects model (LME) in which we controlled for the non-independence of pairwise observations by including as random effects the identities of the two individuals being compared. To test for a relationship between MHC heterozygosity and sMLH, we used a binomial generalised linear model (GLM). Statistical significance was determined using an *F*-test in the LME and a χ^2^-test in the GLM.

### Tests for selection

Nucleotide sequences were aligned to full-length DQB sequences of the California sea lion and trimmed to the reading frame of the second exon with respect to Brown et al.^8^, resulting in 267 bp DQB exon 2 sequences. Rates of synonymous (dS) and non-synonymous (dN) substitutions were estimated in MEGA using the modified Nei–Gojobori model with Jukes-Cantor correction. Rates were calculated for the entire exon as well as separately for the putative antigen-binding sites (pABS; 24 codons) and non-pABS (65 codons). Codons of the pABS were identified with respect to Brown et al.^8^. However, it should be noted that the homology of these putative antigen binding sites among mammalian MHC class II molecules remains hypothetical. The significance of differences was assessed by testing for neutral and positive selection using codon-based Z-tests with 999 bootstrap replicates.

### Phylogenetic reconstruction

MHC class II DQB exon 2 sequences from the Antarctic fur seal, various other pinniped species and the dog (*Canis lupus familiaris*) were aligned using MEGA’s ClustalW algorithm. We then constructed a phylogenetic tree using a maximum likelihood approach with the Jukes-Cantor model for nucleotide substitutions.

### Primer binding site mismatches and allele detection simulation

We compared primer binding site mismatches of the alleles identified in this study to the alleles discovered by Hoelzel et al.^70^. We used different threshold values based on the number of nucleotide mismatches to the primer binding site (maximum Hamming distance) to classify the alleles. In a bootstrapping approach, we randomly sampled empirical genotypes with replacement, ranging from one to 56 samples with 999 replacements each, to calculate the number of detected alleles and their mean and standard deviation within the specified threshold of allowed mismatches.

### Animal ethics

Samples were collected and retained under Scientific Research Permits for the British Antarctic Survey field activities on South Georgia, and in accordance with the Convention on International Trade in Endangered Species of Wild Fauna and Flora (CITES). All field procedures were approved by the British Antarctic Survey Animal Welfare and Ethics Review Body (reference no. PEA6).

## Supplementary Information

Supplementary Information 1.

Supplementary Information 2.

## Data Availability

Microsatellite and MHC class II DQB exon 2 genotypes are available via Zenodo (https://doi.org/10.5281/zenodo.6534955). Sequences of the 19 MHC alleles are available via Genbank (accession numbers ON060886–ON060904).

## Code availability

The code used to analyse the data and accompanying documentation are available as a PDF file written in Rmarkdown (electronic supplementary material, File S1, https://github.com/tebbej/ArGa_MHC_DQB_R).
