# Supplementary material for: Intronic primers reveal unexpectedly high major histocompatibility complex diversity in Antarctic fur seals
Source: Sci Rep. 2022 Oct 26;12:17933. doi: 10.1038/s41598-022-21658-7 (PMC9606363; doi:10.1038/s41598-022-21658-7)
Supplement: Supplementary file 1 — Supplementary Information 1. [file 41598_2022_21658_MOESM1_ESM.docx]

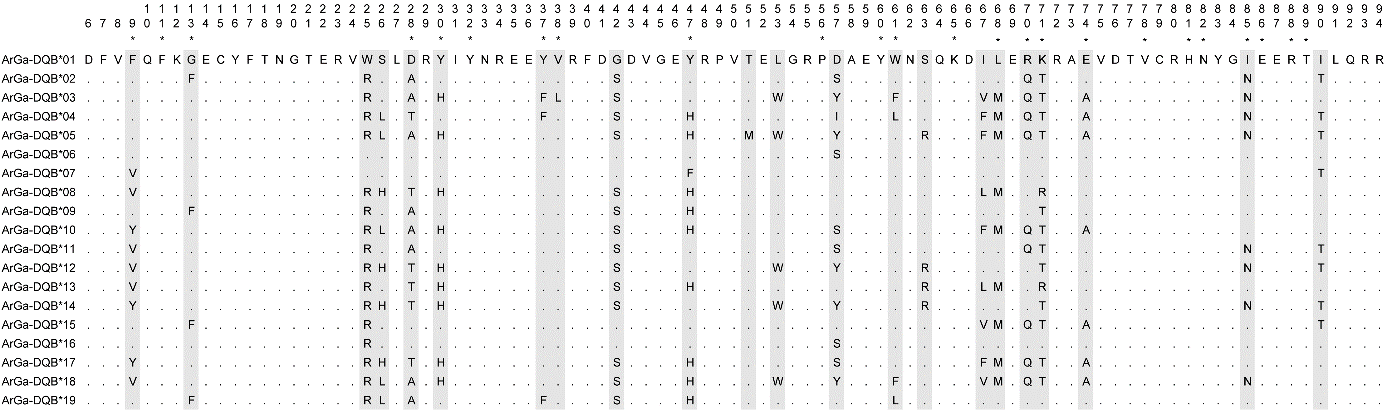
**Supplementary Fig. 1.** ArGa-DQB alleles translated into amino acid sequences. Numbers refer to amino acid positions according to Brown et al. ^8^ and putative antigen binding sites (pABS) are denoted by asterixes. Points indicate identity with ArGa-DQB*01 and variable sites among alleles are shaded in grey.

**
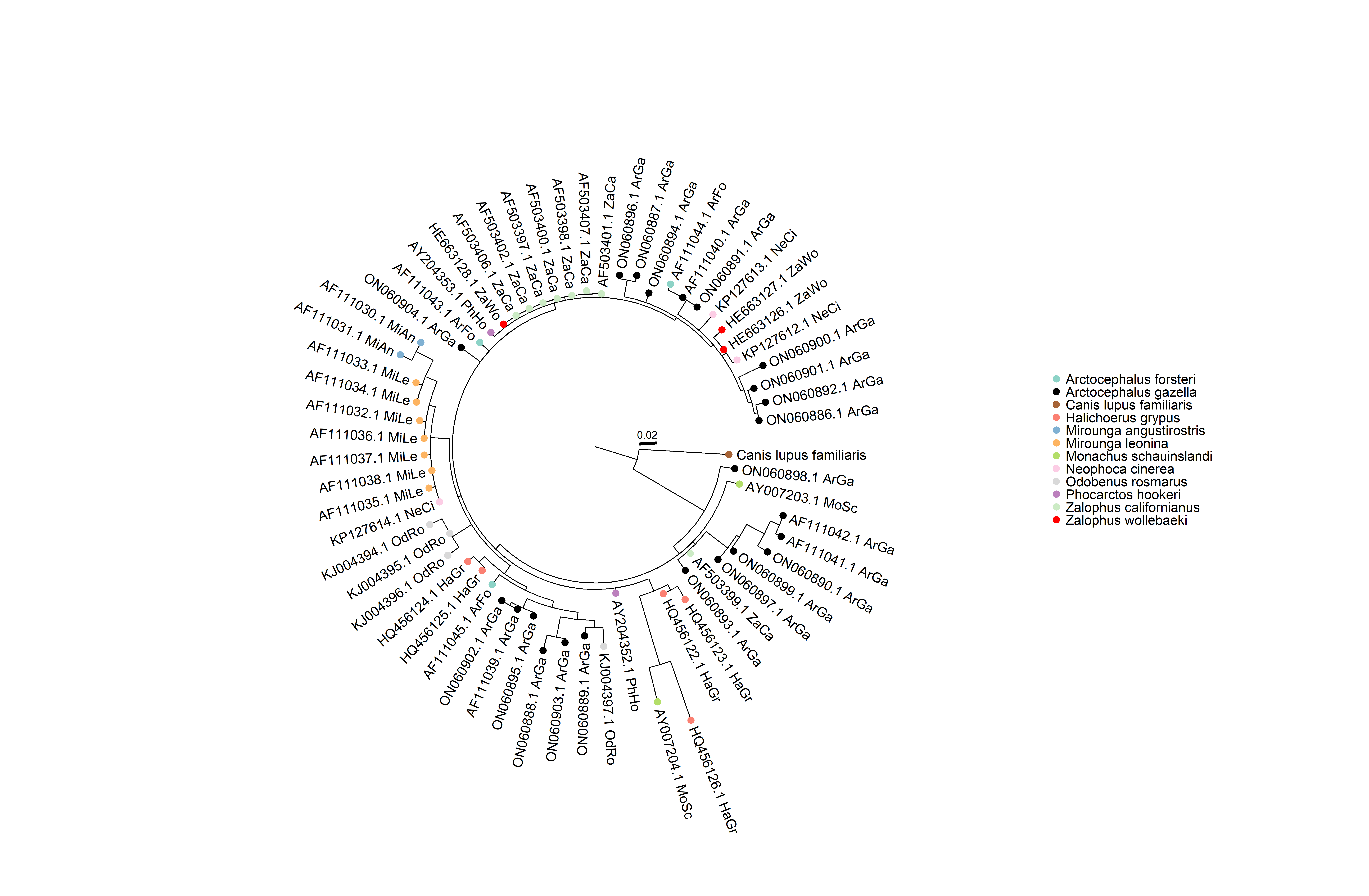
Supplementary Fig. 2.** Phylogenetic reconstruction of MHC class II DQB exon 2 sequences from various pinniped species. The phylogeny is based on a maximum likelihood approach with nearest-neighbour-interchange and a Jukes-Cantor model for nucleotide substitutions. Tip colours represent species affiliations as shown in the legend.


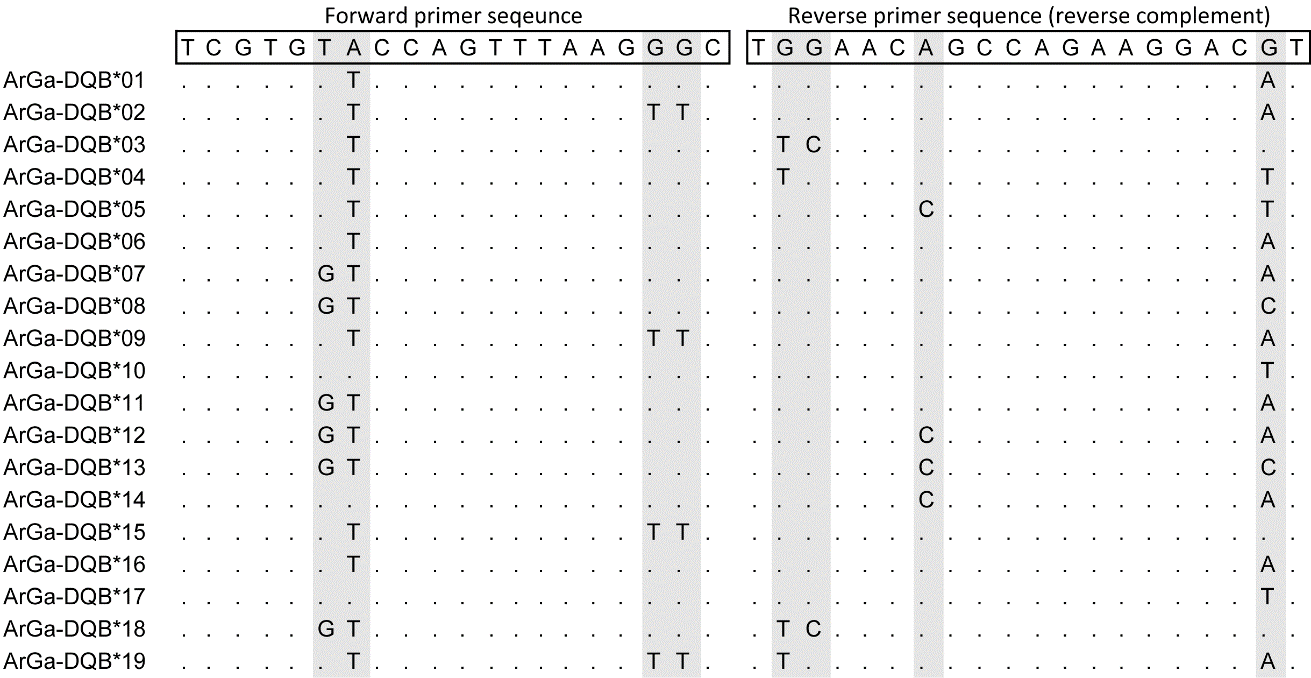
**Supplementary Fig. 3.** Alignment of PCR primers from Hoelzel et al.^70^ and the primer binding sites of 19 ArGa-DQB variants characterised in this study. Identity with primer sequences is shown by points and variable sites are shaded in grey.

**Supplementary table 1.** Summary of the cloning dataset including details of the mother-offspring pairs sampled from both colonies, the number of clone sequences obtained and the inferred genotype of each individual. ‘Clone number’ refers to the number of clones sequenced per individual. The numbers shown in the ‘MHC genotype’ column refer to the ArGa-DQB alleles depicted in Figure 2. Asterisks indicate individuals that were subjected to multiple rounds of cloning. Hyphens refer to individuals within pairs that could not be successfully cloned and sequenced.

| Colony | Mother | | | Pup | | |
| --- | --- | --- | --- | --- | --- | --- |
|  | ID | Clone number | MHC genotype | ID | Clone number | MHC genotype |
| SSB | W8565_mother | – | – | W8565_pup | 11 | 4,7 |
|  | W8568_mother | – | – | W8568_pup | 13 | 3,4 |
|  | W8569_mother* | 26 | 2,10 | W8569_pup | 7 | 8,10 |
|  | W8570_mother | 11 | 5,12 | W8570_pup | 12 | 1,5 |
|  | W8571_mother | – | – | W8571_pup | 12 | 7,7 |
|  | W8572_mother | 9 | 1,15 | W8572_pup | 14 | 1,19 |
|  | W8573_mother | 11 | 8,10 | W8573_pup | 14 | 2,10 |
|  | W8574_mother | 14 | 4,6 | W8574_pup | 12 | 4,9 |
|  | W8576_mother | 13 | 16,16 | W8576_pup | 10 | 8,11 |
|  | W8577_mother | – | – | W8577_pup | 9 | 8,8 |
|  | W8578_mother | 15 | 3,8 | W8578_pup | 15 | 8,10 |
|  | W8580_mother | 12 | 1,13 | W8580_pup | – | – |
|  | W8581_mother | 11 | 1,5 | W8581_pup | – | – |
|  | W8582_mother | 11 | 1,5 | W8582_pup | – | – |
|  | W8583_mother | 13 | 9,9 | W8583_pup | – | – |
|  | W8585_mother | 11 | 1,3 | W8585_pup | – | – |
| FWB | W8552/W8258_mother | 15 | 5,11 | W8552/W8258_pup | – | – |
|  | W8913_mother | 13 | 1,1 | W8913_pup | 15 | 6,6 |
|  | W8914_mother* | 25 | 2,4 | W8914_pup | 12 | 2,9 |
|  | W8915_mother | 10 | 4,15 | W8915_pup | 15 | 9,15 |
|  | W8916_mother | 14 | 8,11 | W8916_pup | – | – |
|  | W8920_mother | 13 | 3,3 | W8920_pup | – | – |
|  | W8921_mother | 13 | 3,3 | W8921_pup | – | – |
|  | W8922_mother | 14 | 7,7 | W8922_pup | 12 | 1,7 |
|  | W8923_mother* | 28 | 1,5,10 | W8923_pup | 14 | 5,11 |
|  | W8924_mother | 12 | 1,4 | W8924_pup | – | – |
|  | W8925_mother | 11 | 6,7 | W8925_pup | 14 | 7,7 |
|  | W8927_mother | 13 | 1,3 | W8927_pup | 12 | 3,12 |
|  | W8928_mother | 13 | 2,13 | W8928_pup | 12 | 1,2 |
|  | W8930_mother | 10 | 2,10 | W8930_pup | 12 | 1,2 |
|  | W8931_mother | 12 | 5,18 | W8931_pup | 13 | 5,11 |
|  | W8933_mother | 12 | 17,17 | W8933_pup* | 32 | 1,15 |
|  | W8935_mother* | 25 | 2,6,14 | W8935_pup | 11 | 2,6 |
|  | W8936_mother | 13 | 1,1 | W8936_pup | – | – |
|  | W8937_mother | 15 | 12,14 | W8937_pup | 14 | 1,14 |
|  | W8939_mother | 15 | 2,9 | W8939_pup | – | – |

**Supplementary table 2.** Genetic diversity statistics for two Antarctic fur seal breeding colonies, the special study beach (SSB) and freshwater beach (FWB). See Materials and methods for details.

| **(a) MHC II DQB exon 2** | | | | | | |
| --- | --- | --- | --- | --- | --- | --- |
|  | **All individuals (*n* = 56)** | | **Mothers only (*n* = 32)** | | **Pups only (*n* = 24)** | |
|  | **SSB** | **FWB** | **SSB** | **FWB** | **SSB** | **FWB** |
| **Private alleles** | 4 | 5 | 2 | 6 | 12 | 7 |
| **Allelic richness** | 16.00 | 15.44 | 14.00 | 13.16 | 11.00 | 10.46 |
| ***H*_obs_** | 0.83 | 0.76 | 0.83 | 0.70 | 0.82 | 0.85 |
| ***H*_exp_** | 0.93 | 0.92 | 0.95 | 0.94 | 0.92 | 0.92 |
| ***F*_is_** | 0.111 | 0.180 | 0.120 | 0.254 | 0.109 | 0.083 |
| ***F*_st_** | 0.0054 | | -0.0212 | | 0.0285 | |
| **(b) Microsatellites** | | | | | | |
|  | **All individuals (*n* = 56)** | | **Mothers only (*n* = 32)** | | **Pups only (*n* = 24)** | |
|  | **SSB** | **FWB** | **SSB** | **FWB** | **SSB** | **FWB** |
| **Private alleles** | 41 | 108 | 34 | 149 | 77 | 102 |
| **Allelic richness** | 7.82 | 7.60 | 6.47 | 6.74 | 6.14 | 6.40 |
| ***H*_obs_** | 0.78 | 0.79 | 0.79 | 0.77 | 0.77 | 0.82 |
| ***H*_exp_** | 0.78 | 0.79 | 0.79 | 0.79 | 0.78 | 0.79 |
| ***F*_is_** | -0.007 | -0.012 | -0.010 | 0.017 | 0.011 | -0.043 |
| ***F*_st_** | 0.0028 | | -0.0081 | | 0.0048 | |

**Supplementary table 3.** Genbank accession numbers of carnivore MHC sequences used to discover the MHC class II DQB exon 2 in the Antarctic fur seal.

| Species | Abbreviation | GenBank accession numbers |
| --- | --- | --- |
| New Zealand fur seal, *Arctocephalus forsteri* | ArFo | AF111043.1–AF111045.1 |
| Antarctic fur seal, *Arctocephalus gazella* | ArGa | AF111039.1–AF111042.1 |
| Grey seal, *Halichoerus grypus* | HaGr | HQ456122–HQ456126 |
| Domestic dog, *Canis lupus familiaris* | CaFa | AF016904.1-AF016909.1 |
| Northern elephant seal, *Mirounga angustirostris* | MiAn | AF111030.1–AF111031.1 |
| Southern elephant seal, *Mirounga leonina* | MiLe | AF111032.1–AF111038.1 |
| Hawaiian monk seal, *Neomonachus schauinslandi* | NeSc | AY007203.1–AY007204.1 |
| Australian sea lion, *Neophoca cinerea* | NeCi | KP127612.1–KP127614.1 |
| Walrus, *Odobenus rosmarus* | OdRo | KJ004394.1–KJ004397.1 |
| New Zealand sea lion, *Phocarctos hookeri* | Phho | AY204352–AY204353 |
| California sea lion, *Zalophus californianus* | ZaCa | AF503397.1–AF503407.1 |
| Galapágos sea lion, *Zalophus wollebaeki* | Zawo | HE663126–HE663128 |
