# Supplementary material for: Intronic primers reveal unexpectedly high major histocompatibility complex diversity in Antarctic fur seals
Source: Sci Rep. 2022 Oct 26;12:17933. doi: 10.1038/s41598-022-21658-7 (PMC9606363; doi:10.1038/s41598-022-21658-7)
Supplement: Supplementary file 2 — Supplementary Information 2. [file 41598_2022_21658_MOESM2_ESM.pdf]

Intronic primers reveal unexpectedly high major histocompatibility complex diversity in Antarctic fur seals - Code

J. Tebbe, M. Ottensmann & J.I. Hoffman

23 August, 2022

This document provides all the `R code` used in Hoffman *et al.* (2022). Both the Rmarkdown file and the data can be downloaded from the accompanying GitHub repository on (https://github.com/tebbej/ArGa_MHC_DQB_R) as a zip archive containing all the files. We recommend to download or clone this GitHub repository in order to access the documentation together with all the files that are needed to repeat analyses shown in this document. Just click on the link above and then on the green box `Clone or download`. In order to function properly, the same structure of folders must be kept. If you have any questions, don't hesitate to contact jonas.tebbe@uni-bielefeld.de

Packages used for analysis

Necessary packages to run this script. Missing packages that are listed on `CRAN` can be installed with `install.packages()`, whereas `phyloseq` is available via the `Bioconductor` project:

```
## Packages used for analyses
## ---------------------------------------------------------------------------
if (!require("ade4", quietly = TRUE)) {
  install.packages("ade4")
  library(ade4)
} else {
library(ade4) # data analysis function
}
if (!require("adegenet", quietly = TRUE)) {
  install.packages("adegenet")
  library(adegenet)
} else {
library(adegenet) # handling genetic data / genind objects
}
if (!require("ape", quietly = TRUE)) {
  install.packages("ape")
  library(ape)
} else {
library(ape) # handling phylogenetic tree data
}
if (!require("BiocManager", quietly = TRUE)){
    install.packages("BiocManager")

BiocManager::install("Biostrings")
library(Biostrings)
} else {
library(Biostrings) # easily work with genetic string sets
}
```

```r
if (!require("Demerelate", quietly = TRUE)) {
  install.packages("Demerelate")
  library(Demerelate)
} else {
library(Demerelate) # easily work with genetic string sets
}
if (!require("EnvStats", quietly = TRUE)) {
  install.packages("EnvStats")
  library(EnvStats)
} else {
library(EnvStats) # environmental statistics
}
if (!require("genepop", quietly = TRUE)) {
  install.packages("genepop")
  library(genepop)
} else {
library(genepop) # population genetic analyses
}
if (!require("hierfstat", quietly = TRUE)) {
  install.packages("hierfstat")
  library(hierfstat)
} else {
library(hierfstat) # hierarchical F-statistics
}
if (!require("inbreedR", quietly = TRUE)) {
  install.packages("inbreedR")
  library(inbreedR)
} else {
library(inbreedR) # population genetic analyses
}
if (!require("lme4", quietly = TRUE)) {
  install.packages("inbreedR")
  library(lme4)
} else {
library(lme4) # population genetic analyses
}
if (!require("phyloseq", quietly = TRUE)) {
  if (!require("BiocManager", quietly = TRUE)) {
    install.packages("BiocManager")
  }
  BiocManager::install(pkgs = "phyloseq")
  library(phyloseq) # phyloseq objects
} else {
  library(phyloseq) # phyloseq objects
}
if (!require("poppr", quietly = TRUE)) {
  install.packages("poppr")
  library(poppr)
} else {
library(poppr) # population genetic analyses
}
if (!require("vegan", quietly = TRUE)) {
  install.packages("vegan")
```

```r
  library(vegan)
} else {
library(vegan) # statistical tools
}

## data/object handling
## ----------------------------------------------------------------------------------
if (!require("tidyverse", quietly = TRUE)) {
  install.packages("tidyverse")
  library(tidyverse)
} else {
library(tidyverse) # package collection for easy and pretty data science with R
}
if (!require("patchwork", quietly = TRUE)) {
  install.packages("patchwork")
  library(patchwork)
} else {
library(patchwork)
}
if (!require("reshape2", quietly = TRUE)) {
  install.packages("reshape2")
  library(reshape2)
} else {
library(reshape2)
}
if (!require("RColorBrewer", quietly = TRUE)) {
  install.packages("RColorBrewer")
  library(RColorBrewer)
} else {
library(RColorBrewer)
}
if (!require("magrittr", quietly = TRUE)) {
  install.packages("magrittr")
  library(magrittr)
} else {
library(magrittr) # pipe operators
}
if (!require("gridExtra", quietly = TRUE)) {
  install.packages("gridExtra")
  library(gridExtra)
} else {
library(gridExtra) # ggplot grid manipulations
}
if (!require("ggpubr", quietly = TRUE)) {
  install.packages("ggpubr")
  library(ggpubr)
} else {
library(ggpubr) # ggplot grid and plot alignment functions
}
if (!require("egg", quietly = TRUE)) {
  install.packages("egg")
  library(egg)
} else {
```

```r
library(egg) # ggplot grid and plot alignment functions
}
if (!require("treeio", quietly = TRUE)) {
  if (!require("BiocManager", quietly = TRUE)) {
    install.packages("BiocManager")
  }
  BiocManager::install(pkgs = "treeio")
  library(treeio) # phylogeny objects
} else {
  library(treeio) # phylogeny objects
}
if (!require("ggtree", quietly = TRUE)) {
  if (!require("BiocManager", quietly = TRUE)) {
    install.packages("BiocManager")
  }
  BiocManager::install(pkgs = "ggtree")
  library(ggtree) # phylogeny plotting
} else {
  library(ggtree) # phylogeny plotting
}
```

Generate data sets for analyses

Main data set is a `.fas-File` containing multiple clone sequences for several individuals of the Antarctic fur seal (*Arctozephalus gazella*). Load in as a DNAStringSet.

```r
## read sequences from FASTA file
## ---------------------------------------------------------------------------
genotype_info <- readDNAStringSet("data/Clones_MHC_ArGa_exon_20210319.fas")

## show first six sequences
## ---------------------------------------------------------------------------
# head(genotype_info)
#DNAStringSet object of length 6:
 #   width seq                                                 names
#[1]   270 AGGATTTCGTGTTCCAGTTTAAGGGCGAGTGCT...GAATGAGGAGAGAACGACCTTGCAGCGGCGAG AGF11008-C1-I_Klo...
#[2]   270 AGGATTTCGTGTTCCAGTTTAAGGGCGAGTGCT...GAATGAGGGGAGAACGACCTTGCAGCGGCGAG AGF11008-C1-I_Klo...
#[3]   270 AGGATTTCGTGTTCCAGTTTAAGGGCGAGTGCT...GAATGAGGAGAGAACGACCTTGCAGCGGCGAG AGF11008-C1-I_Klo...
#[4]   270 AGGATTTCGTGTTCCAGTTTAAGGGCGAGTGCT...GAATGAGGAGAGAACGACCTTGCAGCGGCGAG AGF11008-C1-I_Klo...
#[5]   270 AGGATTTCGTGTTCCAGTTTAAGGGCGAGTGCT...GAATGAGGAGAGAACGACCTTGCAGCGGCGAG AGF11008-C1-I_Klo...
#[6]   270 AGGATTTCGTGTTCCAGTTTAAGGGCGAGTGCT...GAATGAGGAGAGAACGACCTTGCAGCGGCGAG AGF11008-C1-I_Klo...
```

Read in the respective metadata for cloned individuals.

```r
## load meta data and reformat variables
## ---------------------------------------------------------------------------
metadata_df <- read.table(file   = "data/sample_list.txt",
                          header = T) %>%
  mutate(
    real_id  = factor(real_id,
                      levels = str_sort(real_id,
                                        numeric = T)),
    colony   = as.factor(colony),
    maturity = as.factor(maturity),
    family   = as.factor(family)
```

```
    ) %>%
  arrange(real_id) %>%
  arrange(colony)

## show head of data frame
## -------------------------------------------------------------------------------
head(metadata_df)
##          clone_id        real_id colony maturity family
## 1 W8552W8258mum W8552W8258mum    FWB        M      1
## 2      W8913mum      W8913mum    FWB        M      2
## 3      W8913pup      W8913pup    FWB        P      2
## 4      W8914mum      W8914mum    FWB        M      3
## 5      W8914pup      W8914pup    FWB        P      3
## 6      W8915mum      W8915mum    FWB        M      4
## object structure
## -------------------------------------------------------------------------------
str(metadata_df)
## 'data.frame':    56 obs. of  5 variables:
##  $ clone_id: chr  "W8552W8258mum" "W8913mum" "W8913pup" "W8914mum" ...
##  $ real_id : Factor w/ 56 levels "W8552W8258mum",..: 1 25 26 27 28 29 30 31 32 33 ...
##  $ colony  : Factor w/ 2 levels "FWB","SSB": 1 1 1 1 1 1 1 1 1 1 ...
##  $ maturity: Factor w/ 2 levels "M","P": 1 1 2 1 2 1 2 1 1 1 ...
##  $ family  : Factor w/ 36 levels "1","2","3","4",..: 1 2 2 3 3 4 4 5 6 7 ...
```

Subset metadata to handle mother-pup pair analysis more easily

```
## subset data frame to retrieve complete mother-pup pairs
## -------------------------------------------------------------------------------
metadata_df_pairs <- metadata_df[
  which(
    duplicated(metadata_df$family) | duplicated(metadata_df$family, fromLast = T)
    == T)
  ,]
```

Identify the names and index positions of unique sequences (only occurring once throughout the whole sequences data set) in the .fas-file

```
## Define function to identify sequences that are unique
## -------------------------------------------------------------------------------
# Extracts names and index positions of a StringSet of aligned DNA sequences
# raw_clones = StringSet of aligned same length sequences
# allowed.unique.seq = Number of filtered sequences handled as unique
# max.mismatch = Allowed mismatches to identify unique sequences
get_unique_seqs <- function(raw_clones, allowed.unique.seq = 1, max.mismatch = 0){
  ## coerce to vector
  ## -----------------------------------------------------------------------------
  if (is.vector(raw_clones) != T) {
    raw_clones <- as.vector(raw_clones)
  }
  ## sort all instances that only occur once
  ## -----------------------------------------------------------------------------
  unique_indicator <- vector(length = length(raw_clones))
  for (i in seq_along(raw_clones)) {
    # unique_indicator[i] <- sum(match(raw_clones, raw_clones[i]), na.rm = T)
    unique_indicator[i] <- sum(vcountPattern(as.character(raw_clones[i]), raw_clones,
```

```r
                                          max.mismatch = max.mismatch,
                                          min.mismatch = 0)
                             , na.rm = T)
  } #end for i

  ## index of unique seqs
  ## ---------------------------------------------------------------------------
  index_unique_seq <- which(
    unique_indicator == allowed.unique.seq |
      unique_indicator <= allowed.unique.seq
  )

  ## corresponding unique names
  ## ---------------------------------------------------------------------------
  names_unique_seq <- names(raw_clones[index_unique_seq])

  ## return output
  ## ---------------------------------------------------------------------------
  return(list(index_unique_seq = index_unique_seq,
              names_unique_seq = names_unique_seq))
} #end get_unique_seqs
```

Within a DNAStringSet, comprising multiple DNA alignments of same length sequences, find unique sequences (`get_unique_seqs` function) and remove them from the input data. Use the updated input to create a data frame of identified alleles based on unique sequences found in multiple individuals. Alleles are named from most to least abundant.

```r
## Identify and name alleles
## -----------------------------------------------------------------------------
# data = DNAStringSet of same length sequence alignment
# allele_name = Prefix of allele names
# rm.unique = Bool whether single sequence occurrences are removed
get_allele_info <- function(data, allele_name = "ArGa-DQB*", rm.unique = T){
  if (rm.unique == T) {
    unique_identifier <- get_unique_seqs(data,
                                         allowed.unique.seq = 1,
                                         max.mismatch = 0)

    # delete unique sequences from the data
    if (!purrr::is_empty(unique_identifier[[1]])) {
      genotype_fas <- data[-unique_identifier$index_unique_seq]
    }
  } else {
    genotype_fas <- data
  }

  # create data set with allele sequences
  allele_seq <- unique(genotype_fas)

  # count occurrences of identified alleles in the data
  allele_count <- as.vector(
    sapply(seq_along(allele_seq), function(i)
      sum(match(genotype_fas, allele_seq[[i]]), na.rm = T))
  )
```

```r
  # create data.frame where alleles will be named after
  # its decreasing frequency in the data
  # sorted by the prior allele count
  alleles <- data.frame(seq = as.vector(allele_seq),
                        counts = allele_count,
                        row.names = NULL) %>%
    arrange(., desc(counts)) %>%
    mutate(frequency = (counts/sum(counts))*100) %>%
    `rownames<-`(., sapply(seq_along(allele_count), function(i)
      paste0(allele_name,i))) %>%
    rownames_to_column("name")

  out = list(alleles = alleles,
             genotype_fas = genotype_fas)

  return(out)
} # end get_allele_info
```

The output of the function created above is a list of two data frames. Split both to use further on

```r
## Get alleles
## ------------------------------------------------------------------------------
out <- get_allele_info(genotype_info)

##  Split output
## ------------------------------------------------------------------------------
alleles <- out[[1]]
genotype_fas <- out[[2]]
```

Create data frame for each clone with an allele found more than once in the whole clone data .fas-file.

```r
## Data frame based on all clone sequences
## ------------------------------------------------------------------------------
clone_allele_df <- as.data.frame(genotype_fas) %>%
  transmute(sequence = x) %>%
  rownames_to_column(var = "clone_var")

## match clones to corresponding allele sequences
## ------------------------------------------------------------------------------
allele_index_in_df <- as.vector(
  sapply(
    clone_allele_df$sequence,
    function(x) match(x, alleles$seq)))

## Summarise
## ------------------------------------------------------------------------------
clone_allele_df  %<>%
  transmute(.,
            id         = sapply(
              clone_allele_df$clone_var,
              function(x) {
                stringr::str_split(x, "-")[[1]][1] %>%
                  paste0(., collapse = "-") %>%
                  as.factor()
```

```r
              }),
            clone_var  = clone_var,
            allele     = alleles$name[allele_index_in_df],
            variant_no = allele_index_in_df,
            variant_count  = alleles$counts[allele_index_in_df],
            sequence   = sequence
  ) %>%
  mutate(., allele = factor(
    allele,
    levels = str_sort(
      unique(allele),
      numeric = T))
  ) %>%
  arrange(
    ., allele
  ) %>%
  mutate(
    variant_counter = as.vector(
      unlist(
        sapply(alleles$counts,
               function(x) seq(1:x))
      ))
  ) %>%
  relocate(., sequence, .after = last_col())

## Update clone_allele_df with metadata information
## Summarise sequences
## ----------------------------------------------------------------------------
# create index vector where sample ids correspond to the correct
# names in the metadata data.frame
index <- match(as.character(clone_allele_df$id), metadata_df$clone_id)

# rearrange columns in clone_allele_df based on 'index'
# -----------------------------------------------------------------------------
clone_allele_df <- clone_allele_df %>%
  mutate(
    id       = metadata_df$real_id[index],
    colony   = metadata_df$colony[index],
    maturity = metadata_df$maturity[index],
    family   = metadata_df$family[index]
  ) %>%
  relocate(., sequence, .after = last_col())
clone_allele_df  %<>% arrange(., variant_no)

# clone_allele_df
# $ id              : sample individual
# $ clone_var       : clone sample
# $ allele          : allele as name
# $ variant_no      : allele as number
# $ variant_count   : allele total count
# $ variant_counter : allele counter; 1:last number of occurrence per allele
# $ colony          : colony tag
# $ maturity        : age by maturity, either mother (M) or pup (P)
```

```
# $ sequence        : MHC DQB class II exon sequence of clone_var
```

`clone_allele_df` contains information for clone variants for 56 sample individuals. Data frame structure includes original DNA sample, allele name, stratum data for allele names and individual meta data corresponding to `metadata_df`.

```
str(clone_allele_df, vec.len = 0)
## 'data.frame':    793 obs. of  10 variables:
##  $ id             : Factor w/ 56 levels "W8552W8258mum",..: NULL ...
##  $ clone_var      : chr   ...
##  $ allele         : Factor w/ 30 levels "ArGa-DQB*1","ArGa-DQB*2",..: NULL ...
##  $ variant_no     : int  NULL ...
##  $ variant_count  : int  NULL ...
##  $ variant_counter: int  NULL ...
##  $ colony         : Factor w/ 2 levels "FWB","SSB": NULL ...
##  $ maturity       : Factor w/ 2 levels "M","P": NULL ...
##  $ family         : Factor w/ 36 levels "1","2","3","4",..: NULL ...
##  $ sequence       : chr   ...
```

Create a suitable data frame for a heatmap that contains allele names, sample ids and the respective number an allele occurs in sample id

```
## Summarise alleles
## ---------------------------------------------------------------------------
allele_summary <- matrix(nrow = length(unique(clone_allele_df$id)),
                         ncol = length(unique(clone_allele_df$allele))) %>%
  `rownames<-`(., as.character(unique(clone_allele_df$id))) %>%
  `colnames<-`(., as.character(
    str_sort(
      levels(
        clone_allele_df$allele),
      numeric = T)))

## Fill matrix with info on which and how many alleles are found in the
## clones for each individual fur seal
## Summarise sequences
## ---------------------------------------------------------------------------
for (i in seq_along(unique(clone_allele_df$id))) {
  alleles_in_id <- summary(
    clone_allele_df$allele[clone_allele_df$id == unique(clone_allele_df$id)[i]]
    )
  allele_summary[i, ] <- alleles_in_id[str_sort(names(alleles_in_id),
                                                 numeric = T)]
}

## convert to data.frame and create a "tidy" version
## ---------------------------------------------------------------------------
allele_summary %<>%
  t() %>%
  as.data.frame() %>%
  rownames_to_column("alleles") %>%
  pivot_longer(-c(alleles),
               names_to = "sample_id",
               values_to = "counts") %>%
  mutate(., alleles = factor(
```

```
    alleles,
    levels = str_sort(
      unique(alleles),
      numeric = T)),
    sample_id = as.factor(sample_id)) %>%
  arrange(., sample_id) %>%
  arrange(., alleles)

index2 <- match(as.character(allele_summary$sample_id), metadata_df$real_id)
allele_summary <- allele_summary %>%
  mutate(colony = metadata_df$colony[index2],
         maturity = metadata_df$maturity[index2],
         family = metadata_df$family[index2],
         sample_id = factor(sample_id,
                            levels = rev(
                              levels(sample_id)))) %>%
  arrange(desc(sample_id)) %>%
  arrange(alleles)

pair_match_index <- match(allele_summary$sample_id, metadata_df_pairs$real_id)
pair_match_index <- which(is.na(pair_match_index) == T)
allele_summaryX <- allele_summary[-pair_match_index,]

allele_summaryX <- allele_summaryX[allele_summaryX$alleles %in%
                                     levels(allele_summaryX$alleles)[1:19],]
```

Create data.frame with genotype information based on 19 alleles

```
## Retrieve genotypes
## ----------------------------------------------------------------------------
clone_genotype_df <- allele_summary[
  allele_summary$alleles %in% levels(allele_summary$alleles)[1:19],]

## Eliminate alleles that were not scored in a given individual
## ----------------------------------------------------------------------------
clone_genotype_df <- clone_genotype_df[which(clone_genotype_df$counts != 0),]

## Define small helper function
## ----------------------------------------------------------------------------
f1 <- function(x){
  length(
    na.omit(
      match(clone_genotype_df$alleles, x)
    ))
}

clone_genotype_df  %<>%
  mutate(.,
         variant_no = clone_allele_df$variant_no[
           match(clone_genotype_df$alleles,
                 clone_allele_df$allele)],
         freq = clone_allele_df$allele_frequency[
           match(clone_genotype_df$alleles,
                 clone_allele_df$allele)],
```

```
  ) %>%
  arrange(., alleles) %>%
  mutate(.,
         variant_counts = unlist(
           sapply(clone_genotype_df$alleles, f1))) %>%
  mutate(.,
         variant_counter = unlist(
           sapply(
             sapply(
               unique(
                 clone_genotype_df$alleles), f1),
             function(x) seq(1:x)))) %>%
  arrange(., desc(variant_counter))
```

Create list for figure storing

```
figures <- vector(mode = "list")
```

Plot clone sequence frequencies

```
## index to remove putative artefacts
## ---------------------------------------------------------------------------
figures[[1]] <- ggplot(clone_allele_df[1:771,],
                       aes(x = variant_no,
                           group = dplyr::desc(variant_counter),
                           fill = variant_counter)) +
  geom_bar(aes(y = stat(count) / sum(count))) +
  scale_y_continuous(labels = scales::label_percent(accuracy = 1),
                     limits = c(0,.2),
                     expand = c(0,0)) +
  scale_fill_viridis_c(option = "viridis",
                       begin = 0,
                       end = 1) +
  ylab("Frequency\n") +
  labs(fill = "No. of\nclones") +
  scale_x_continuous(name = "MHC DQB II allele",
                     breaks = seq_along(unique(clone_allele_df$allele)),
                     labels = str_sort(unique(clone_allele_df$allele), numeric = T),
                     expand = c(0, 0.3)) +
  theme_minimal() +
  theme(panel.grid = element_line(color = "white"),
        panel.grid.minor = element_blank(),
        panel.grid.major.x = element_blank(),
        axis.line = element_line(color = "black"),
        axis.text = element_text(color = "black"),
        axis.title = element_text(color = "black",
                                  margin = margin(10,10,20,10)),
        axis.ticks = element_line(color = "black",
                                  size = 0.2),
        axis.line.x = element_line(color = "black"),
        axis.ticks.x = element_blank(),
        axis.title.x = element_text(color = "black", size = 15.5),
        axis.text.x.bottom = element_text(angle = 45,
                                          vjust = 1,
```

```
                                          hjust = 1,
                                          size = 13),
        axis.line.y = element_line(color = "black"),
        axis.title.y = element_text(size = 15.5),
        axis.ticks.y = element_blank(),
        axis.text.y.left = element_text(size = 13),
        axis.ticks.length = unit(.15,"cm"),
        plot.background = element_rect(color = "white",
                                       fill = "white"),
        legend.position = c(0.88,.7),
        legend.background = element_rect(fill = "white",
                                         color = "black"),
        plot.margin = unit(c(0.5,0.5,0.5,0,5), "cm")
  )
names(figures)[1] <- "allele_freq_hist"
```

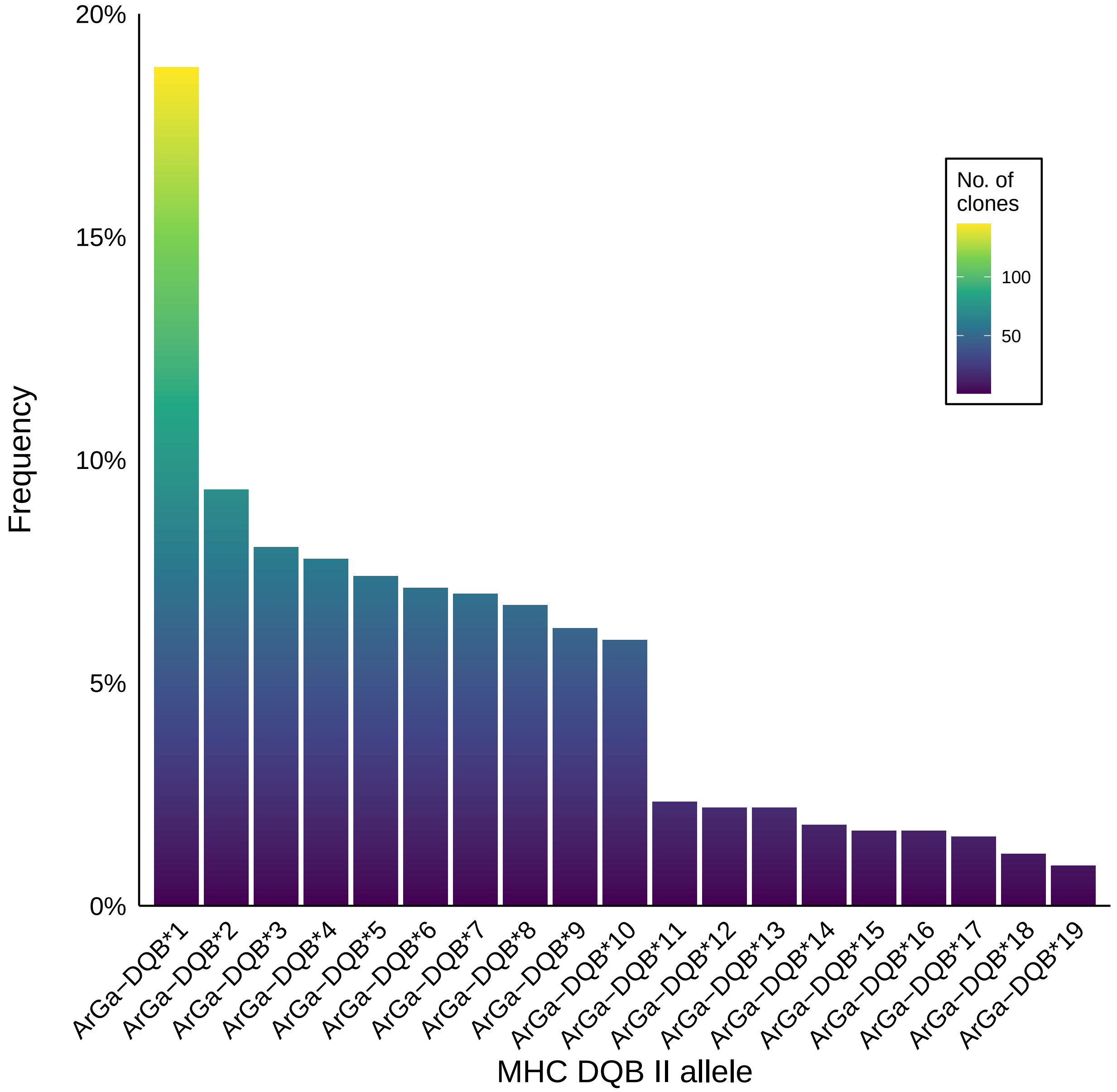

Plot genotype heatmap of mother-pup pairs

```
## plot multiple small heatmaps for single mum-pup pairs
## ---------------------------------------------------------------------------
sep_heat <- function(x, color = "blue"){
  df <- allele_summaryX[which(allele_summaryX$family == x),]
  gg <- ggplot( data = df,
                aes(x = alleles,
                    y = sample_id,
                    fill = log(counts + 1))) +
    geom_tile() +
    coord_fixed(ratio = 0.6) +
    scale_fill_viridis_c(limits = c(0, 3.5),
```

```r
                        begin = 0.05,
                        breaks = 0:3,
                        labels = c(0, 2.7, 7.4, 20.1)) +
    # xlab("Alleles") +
    # ylab("SSB\n") +
    labs(fill = "Log \nclone \nnumber") +
    # theme_minimal() +
    theme(
      panel.grid = element_blank(),
      panel.background = element_blank(),
      axis.text = element_text(color = "black"),
      axis.text.x.bottom = element_blank(),
      axis.text.y = element_text(size = 10,
                                 hjust = 0),
      axis.title.x = element_blank(),
      axis.ticks.x = element_blank(),
      axis.line.y.left = element_line(color = color,
                                      size = 1),
      axis.title.y = element_blank(),
      axis.ticks.y = element_blank(),
      # axis.text.y  = element_text(color = color_ssb),
      plot.margin  = unit(c(-.1, 0, 0, 0), "cm"),
      legend.position = "none"
    )

  return(gg)

}

vec <- unique(allele_summaryX$family)
vec_ssb <- unique(allele_summaryX$family[allele_summaryX$colony == "SSB"])
vec_fwb <- unique(allele_summaryX$family[allele_summaryX$colony == "FWB"])

## create two plot lists for small heatmaps to separate the two colonies
## ----------------------------------------------------------------------------
plots_ssb <- lapply(vec_ssb, sep_heat, color = "white")
plots_fwb <- lapply(vec_fwb, sep_heat, color = "white")

## merge in order
## ----------------------------------------------------------------------------
plot_list <- c(plots_ssb, plots_fwb)

## plot heatmap for last individual with x axis
## ----------------------------------------------------------------------------
l <- length(vec)
plot_list[[l]] <- ggplot(
  data = allele_summaryX[which(allele_summaryX$family == vec[l]),],
  aes(x = alleles,
      y = sample_id,
      fill = log(counts + 1))) +
  geom_tile() +
  coord_fixed(ratio = 0.6) +
  scale_fill_viridis_c(name = "No. of\nclones",
```

```
                      limits = c(0,3.5),
                      begin = 0.05,
                      breaks = 0:3,
                      labels = c(0, 2.7, 7.4, 20.1)) + #log scale
  xlab("Alleles") +
  ylab("FWB\n") +
  # labs(fill = "Log \nclone \nnumber") +
  # theme_minimal() +
  theme(
    panel.grid = element_blank(),
    panel.background = element_blank(),
    axis.ticks = element_line(color = "#000000"),
    axis.text = element_text(color = "black"),
    axis.text.y = element_text(size = 10,
                              hjust = 0),
    axis.title.y = element_blank(),
    axis.ticks.y = element_blank(),
    axis.line.y.left = element_line(color = "white",
                                   size = 1),
    axis.text.x.bottom = element_text(angle = 45,
                                     vjust = 1,
                                     hjust = 1,
                                     size = 10),
    axis.title.x = element_text(size = 14),
    plot.margin = unit(c(-1, 0, 0, 0), "mm"),
    legend.position = "right",
    legend.background = element_rect(fill = "white",
                                    color = "black")
  )

# increase headspace for first list element
## ----------------------------------------------------------------------------
plot_list[[1]] <- plot_list[[1]] + theme(plot.margin = unit(c(10,0,0,0), "mm"))
figures[[2]] <- egg::ggarrange(plots = plot_list, ncol = 1)
```

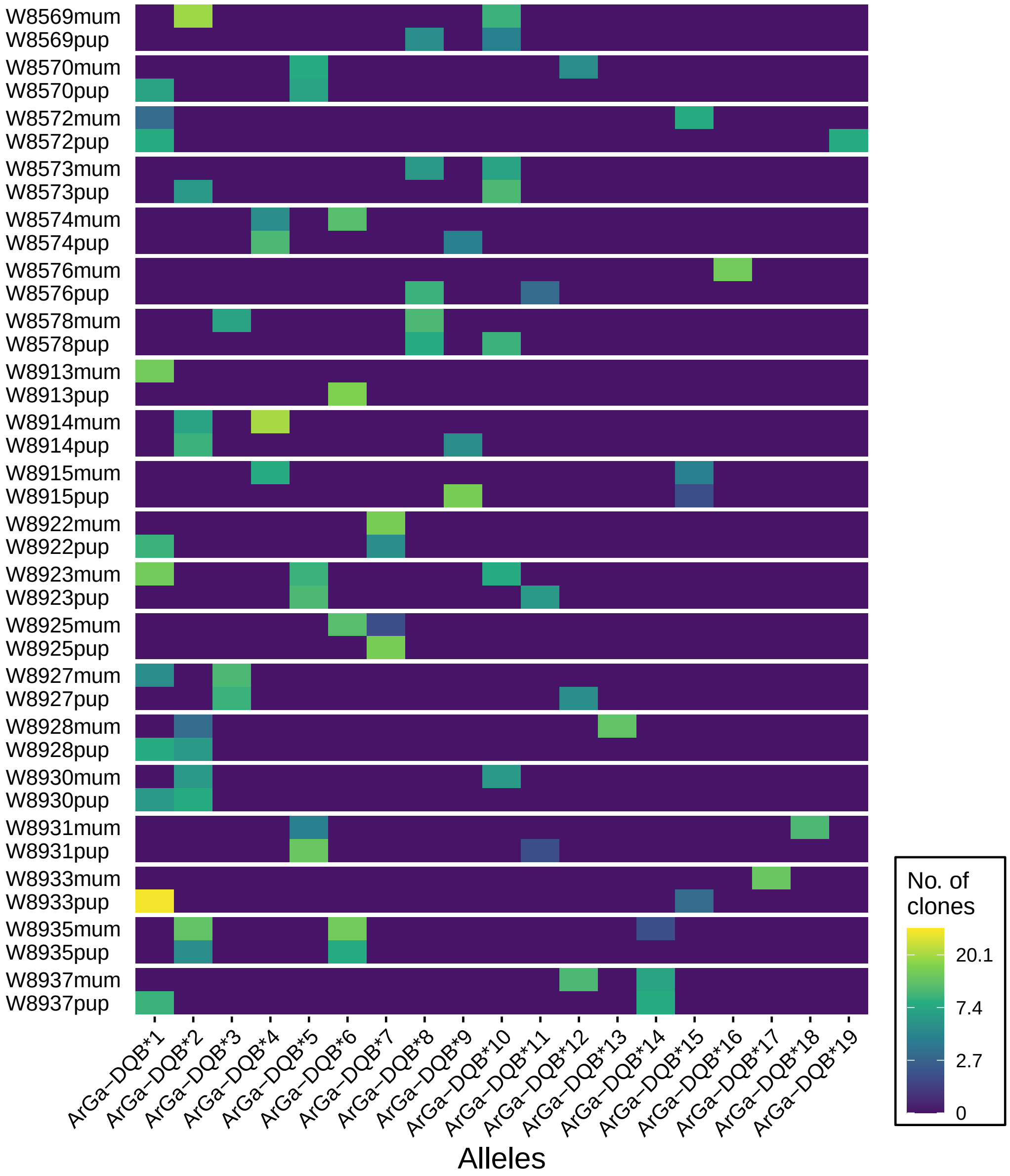


```r
names(figures)[2] <- "clone_heatmap"
```

Colony comparisons

Create `genind` object for easy data handling

Load in genetic data frames and convert to `adegenet`’s Formal `genind` class to easily handle genetic analyses in `R`.
Start with converting genotypes of Antarctic fur seal based on 41 microsatellite loci.

```r
## read microsat data
## ---------------------------------------------------------------------------
msats_gp <- read.table(file = "data/msats_genind.txt", sep = "\t")

# `df2genind` needs input for pop argument
```

```r
sites_msats <- msats_gp[,1]

## only data with genotype info can be converted to genind. Keep original data frame
## ----------------------------------------------------------------------------
msats_gen <- msats_gp[,-1]
msats_gen <- df2genind(msats_gen,
                       ploidy = 2,
                       sep = "/", # alleles are separated with "/"
                       NA.char = NA, # missing loci are NA
                       pop = sites_msats)
```

Convert MHC class II DQB exon 2 genotypes by creating the `genind` object first.

```r
## exclude putative artefacts from main data.frame
## ----------------------------------------------------------------------------
clone_allele_df <- clone_allele_df[1:771,] %>%
  mutate(., variant_no = str_pad(variant_no, 2, pad = "0"))

## create a list of genotypes
## ----------------------------------------------------------------------------
called_clones <- vector(mode = "list", length = 1)
called_clones[[1]] <- seq_along(unlist(attributes(clone_allele_df$id)[1]))
names(called_clones[[1]]) <- unlist(attributes(clone_allele_df$id)[1])


id <- as.character(unique(clone_allele_df$id))
called_clones <- lapply(id, function(x)
  as.character(
    unique(
      clone_allele_df$variant_no[which(!is.na(match(clone_allele_df$id,x)))]
      )
    )
  ) %>%
  `names<-`(., id)

# filter out individuals that do not fit the presumed ploidy of the genotyped locus
# by deleting the least likely allele as we assume diploidy
## ----------------------------------------------------------------------------
ploidy_mismatches <- which(lapply(called_clones, length) > 2)
called_clones[ploidy_mismatches] <- lapply(called_clones[ploidy_mismatches],
                                           function(x) x[1:2])

called_clones <- lapply(called_clones, function(x){
  c(x[1], tail(x,1))
})

# build a data frame like
#                  locusA locusB locusC
#       genotype1     11    <NA>     22
#       genotype2     11      34     22
#       genotype3     12      55     21
#       genotype4     32      15     22
# that can be coerced into a "genind"
clone_df <- lapply(called_clones, function(x)
```

```r
  paste0(x, collapse = "/")) %>%
  as.data.frame(.) %>%
  t(.)


## build data frame with additional info for strata in genind class object
## ----------------------------------------------------------------------------
n <- rownames(clone_df)
ind_n <- match(n,clone_genotype_df$sample_id)
strata_df <- data.frame(
  id   = n,
  pops = clone_genotype_df$colony[ind_n],
  mtry = clone_genotype_df$maturity[ind_n],
  fmly = clone_genotype_df$family[ind_n])


## coerce to genind
## ----------------------------------------------------------------------------
clone_gen <- df2genind(clone_df,
                       ploidy = 2,
                       sep = "/",
                       pop = strata_df$pops,
                       strata = strata_df)

## Save as txt file
## ----------------------------------------------------------------------------
# save.df <- genind2df(clone_gen, sep = "/")
# write.table(save.df, file = "data/clone_gen.txt", sep = "\t")
```

We can use the resulting object as is and save it as a data frame that is easily convertible to a `genind` for future use.
Load in MHC DQB class 2 genotype by data frame

```r
## read MHC class II DQB exon 2 genotyping data
## ----------------------------------------------------------------------------
clone_gp <- read.table(file = "data/clone_genind.txt", sep = "\t")

# `df2genind` needs input for pop argument
sites_clones <- clone_gp[,1]

# 1 column is forced to a vector but must not be a vector to be coerced to
# genind object
clone_gen <- clone_gp[,-1] %>% as.data.frame() %>%
  # keep row and column names
  `rownames<-`(rownames(clone_gp)) %>%
  `colnames<-`("dqbII")

clone_gen <- df2genind(clone_gen,
                       ploidy = 2,
                       sep = "/", # separate alleles with "/"
                       pop = sites_clones)
```

Calculate Fst

Calculate fixation index after Weir & Cockerham (1984)

```
(fst_msats_gen <- genet.dist(msats_gen, method = "WC"))
##               FWB
## SSB 0.002807775
(fst_clone_gen <- genet.dist(clone_gen, method = "WC"))
##               FWB
## SSB 0.005438056
```

Permute to get *p*-values

Permutations are inefficient, thus lowered for demonstration. To get similar results as depicted in the publication, run the following code:

```
set.seed(111)
perm.fst(msats_gen, nperm = 9999)
perm.fst(clone_gen, nperm = 9999)
```

```
## Create function to run permutations
## ----------------------------------------------------------------------------
# `data` = Formal class genind to be permuted for Fst
# `nperm` = number of Fst permutations
# `resamples`  = number of data permutations
perm.fst <- function(data, nperm = 100, resamples = 50){
  x <- data
  df <- genind2df(x, sep = "/")
  resamples <- resamples

  # calculate pairwise fst
  mat.obs <- genet.dist(x, method = "WC") %>% as.matrix()
  mat.obs <- mat.obs[1,2]
  cat("Fst: ", mat.obs, "\n")

  # calculate permute fst
  nperm <- nperm
  mat.perm <- sapply(1:nperm,
                     function(i) {
    # permute
    x <- df[sample(1:nrow(df), resamples), ]
    x <- df2genind(as.matrix(x[,-1]), sep = "/", pop = x[,1])

    # calculate pairwise fst
    mat.fst <- genet.dist(x, method = "WC") %>% as.matrix()
    mat.fst <- mat.fst[1,2]
  })

  # handle as randtest for monte-carlo like simulation of p-values
  test.rand <- as.randtest(
    sim = na.omit(sapply(1:nperm,
                         function(i) mat.perm[i])),
    obs = mat.obs,
    alter = "greater" # ((# of permutations >= mat.obs) + 1) / (# of nperm +1)
    )

  return(test.rand)
```

```
} # end perm.fst

perm.fst(msats_gen)
## Fst:  0.002807775
## Monte-Carlo test
## Call: as.randtest(sim = na.omit(sapply(1:nperm, function(i) mat.perm[i])),
##     obs = mat.obs, alter = "greater")
##
## Observation: 0.002807775
##
## Based on 100 replicates
## Simulated p-value: 0.4851485
## Alternative hypothesis: greater
##
##      Std.Obs  Expectation     Variance
## 5.484944e-02 2.741834e-03 1.445340e-06
perm.fst(clone_gen)
## Fst:  0.005438056
## Monte-Carlo test
## Call: as.randtest(sim = na.omit(sapply(1:nperm, function(i) mat.perm[i])),
##     obs = mat.obs, alter = "greater")
##
## Observation: 0.005438056
##
## Based on 100 replicates
## Simulated p-value: 0.4554455
## Alternative hypothesis: greater
##
##      Std.Obs  Expectation     Variance
## 1.992634e-01 4.493488e-03 2.247044e-05
```

Private alleles per site

```
# msats
private_alleles(msats_gen) %>% apply(MARGIN = 1, FUN = sum)
## FWB SSB
## 108  41
# mhc clones
private_alleles(clone_gen) %>% apply(MARGIN = 1, FUN = sum)
## FWB SSB
##   5   3
```

Allelic richness per site

```
# msats
allelic.richness(genind2hierfstat(msats_gen))$Ar %>%
  apply(MARGIN = 2, FUN = mean) %>%
  round(digits = 3)
##   FWB   SSB
## 7.959 7.819
# mhc clones
allelic.richness(genind2hierfstat(clone_gen))$Ar %>%
  apply(MARGIN = 2, FUN = mean) %>%
```

```
  round(digits = 3)
##    FWB    SSB
## 15.436 16.000
```

Summary of basic statistics per site per locus

```
# msats
msats_gen_stats <- basic.stats(msats_gen, diploid = TRUE)

# mhc clones
clone_gen_stats <- basic.stats(clone_gen, diploid = TRUE)
```

Mean observed heterozygosity per site

```
# msats
apply(msats_gen_stats$Ho, MARGIN = 2, FUN = mean, na.rm = TRUE)
##       FWB       SSB
## 0.7921805 0.7819756
# mhc clone
apply(clone_gen_stats$Ho, MARGIN = 2, FUN = mean, na.rm = TRUE)
##    FWB    SSB
## 0.7576 0.8261
```

Mean expected heterozygosity per site

```
# msats
apply(msats_gen_stats$Hs, MARGIN = 2, FUN = mean, na.rm = TRUE)
##       FWB       SSB
## 0.7860390 0.7795317
# mhc clones
apply(clone_gen_stats$Hs, MARGIN = 2, FUN = mean, na.rm = TRUE)
##    FWB    SSB
## 0.9233 0.9298
```

Inbreeding coefficient FIS

```
# msats
apply(msats_gen_stats$Fis, MARGIN = 2, FUN = mean, na.rm = TRUE)
##          FWB          SSB
## -0.012095122 -0.006690244
# mhc clones
apply(clone_gen_stats$Fis, MARGIN = 2, FUN = mean, na.rm = TRUE)
##    FWB    SSB
## 0.1795 0.1116
```

Repeat analysis. Run mothers and pups separate

```
df.sats <- genind2df(msats_gen, sep = "/")
df.sats <- df.sats[match(metadata_df$real_id, rownames(df.sats)),]

df.mom <- df.sats[which(metadata_df$maturity == "M"),]
df.mom <- df2genind(df.mom[,-1], sep = "/", pop = df.mom[,1])
```

```r
df.pup <- df.sats[which(metadata_df$maturity == "P"),]
df.pup <- df2genind(df.pup[,-1], sep = "/", pop = df.pup[,1])
```

Microsatellite data: Separate mother and pup data

```r
private_alleles(df.mom) %>% apply(MARGIN = 1, FUN = sum)
## FWB SSB
## 149  34
# allelic richness per site (per locus)
allelic.richness(genind2hierfstat(df.mom))$Ar %>%
  apply(MARGIN = 2, FUN = mean) %>%
  round(digits = 3)
##   FWB   SSB
## 6.737 6.474
# all kinds of basic stats
df.mom_stats <- basic.stats(df.mom, diploid = TRUE)

# mean observed heterozygosity per site
apply(df.mom_stats$Ho, MARGIN = 2, FUN = mean, na.rm = TRUE)
##       FWB       SSB
## 0.7708488 0.7887829
# mean expected heterozygosity per site
apply(df.mom_stats$Hs, MARGIN = 2, FUN = mean, na.rm = TRUE)
##       FWB       SSB
## 0.7904512 0.7895707
# inbreeding coefficient F_IS
apply(df.mom_stats$Fis, MARGIN = 2, FUN = mean, na.rm = TRUE)
##          FWB          SSB
##  0.016960976 -0.009217073
# pairwise F_st
genet.dist(df.mom, method = "WC")
##               FWB
## SSB -0.008140639
```

Microsatellite data: Mothers

```r
# private alleles per site (per locus)
private_alleles(df.pup) %>% apply(MARGIN = 1, FUN = sum)
## FWB SSB
## 102  77
# allelic richness per site (per locus)
allelic.richness(genind2hierfstat(df.pup))$Ar %>%
  apply(MARGIN = 2, FUN = mean) %>%
  round(digits = 3)
##   FWB   SSB
## 6.397 6.414
# all kinds of basic stats
df.pup_stats <- basic.stats(df.pup, diploid = TRUE)

# mean observed heterozygosity per site
apply(df.pup_stats$Ho, MARGIN = 2, FUN = mean, na.rm = TRUE)
```

```
##       FWB       SSB
## 0.8251146 0.7746878
# mean expected heterozygosity per site
apply(df.pup_stats$Hs, MARGIN = 2, FUN = mean, na.rm = TRUE)
##       FWB       SSB
## 0.7915415 0.7805878
# inbreeding coefficient F_IS
apply(df.pup_stats$Fis, MARGIN = 2, FUN = mean, na.rm = TRUE)
##         FWB         SSB
## -0.04271220  0.01067805
# pairwise F_st
genet.dist(df.pup, method = "WC")
##             FWB
## SSB 0.004752703
```

Microsatellite data: Pups

```
df.clone <- genind2df(clone_gen, sep = "/")
df.clone <- df.clone[match(metadata_df$real_id, rownames(df.clone)),]

df.mom <- df.clone[which(metadata_df$maturity == "M"),]
df.mom <- df2genind(as.matrix(df.mom[,-1]), sep = "/", pop = df.mom[,1])

df.pup <- df.clone[which(metadata_df$maturity == "P"),]
df.pup <- df2genind(as.matrix(df.pup[,-1]), sep = "/", pop = df.pup[,1])
```

MHC DQB class II data: Separate mother and pup data

```
# private alleles per site (per locus)
private_alleles(df.mom) %>% apply(MARGIN = 1, FUN = sum)
## FWB SSB
##   6   2
# allelic richness per site (per locus)
allelic.richness(genind2hierfstat(df.mom))$Ar %>%
  apply(MARGIN = 2, FUN = mean) %>%
  round(digits = 3)
##    FWB    SSB
## 13.155 14.000
# all kinds of basic stats
df.mom_stats <- basic.stats(df.mom, diploid = TRUE)

# mean observed heterozygosity per site
apply(df.mom_stats$Ho, MARGIN = 2, FUN = mean, na.rm = TRUE)
##    FWB    SSB
## 0.7000 0.8333
# mean expected heterozygosity per site
apply(df.mom_stats$Hs, MARGIN = 2, FUN = mean, na.rm = TRUE)
##    FWB    SSB
## 0.9382 0.9470
# inbreeding coefficient F_IS
apply(df.mom_stats$Fis, MARGIN = 2, FUN = mean, na.rm = TRUE)
##    FWB    SSB
```

```r
## 0.2539 0.1200
# pairwise F_st
genet.dist(df.mom, method = "WC")
##              FWB
## SSB -0.02125225
```

MHC DQB class II data: Mothers

```r

# private alleles per site (per locus)
private_alleles(df.pup) %>% apply(MARGIN = 1, FUN = sum)
## FWB SSB
##   7  12
# allelic richness per site (per locus)
allelic.richness(genind2hierfstat(df.pup))$Ar %>%
  apply(MARGIN = 2, FUN = mean) %>%
  round(digits = 3)
##    FWB    SSB
## 10.461 11.000
# all kinds of basic stats
df.pup_stats <- basic.stats(df.pup, diploid = TRUE)

# mean observed heterozygosity per site
apply(df.pup_stats$Ho, MARGIN = 2, FUN = mean, na.rm = TRUE)
##    FWB    SSB
## 0.8462 0.8182
# mean expected heterozygosity per site
apply(df.pup_stats$Hs, MARGIN = 2, FUN = mean, na.rm = TRUE)
##    FWB    SSB
## 0.9231 0.9182
# inbreeding coefficient F_IS
apply(df.pup_stats$Fis, MARGIN = 2, FUN = mean, na.rm = TRUE)
##    FWB    SSB
## 0.0833 0.1089
# pairwise F_st
genet.dist(df.pup, method = "WC")
##             FWB
## SSB 0.02847633
```

MHC DQB class II data: Pups

Correlate genetic diversity

Correlate measurements for genetic diversity of 41 microsatellite loci and one MHC class II DQB exon 2 locus

Generate genetic diversity estimates

For this study, we base genetic diversity on pairwise genetic distance measurements

Absolute genetic distance: Number of allelic differences in Microsatellite data

Calculate microsatellite relatedness values

```r
# read in genotype data table
msats_df <- read.table("data/msats/msats_genotypes_inbreedR.txt", sep = "\t")

# update data.frame with additional info
# "delete" colony info, otherwise relatedness is only calculated for individuals
# within their own colonies -> no complete pairwise comparison
msats_df <- cbind(id = as.factor(rownames(msats_df)),
                  # colony = metadata_df$colony,
                  colony = rep("col", 56),
                  msats_df[1:56,]) %>%
  # clear df from rownames/ only keep colnames/ variable names
  `rownames<-`(NULL)

msats_df[is.na(msats_df)] = 0

str(msats_df)

write.table(msats_df, file = "data/msats_genotypes_demerelate.txt",
            sep = "\t",
            row.names = F)
```

create data.frame in correspondence to Demerelate input format

```r
relatedness_results <- Demerelate(inputdata = msats_df,
                                  value = "rxy",
                                  object = T,
                                  NA.rm = F,
                                  Fis = F)
```

Calculate relatedness of individuals based on Queller & Goodnight

```r
relatedness <- unlist(relatedness_results$Empirical_Relatedness)

## fill distant matrix / make sure that it follows same systematics as previous distance matrices
## create empty matrix with equal rows and cols similar to sample size of indidivuals
relate_mat <- matrix(nrow = 56, ncol = 56)
## fill distance matrix row wise, thus fill upper.tri
relate_mat[upper.tri(relate_mat)] <- relatedness
## transpose to keep consistency with other distance matrices
relate_mat <- t(relate_mat)
relate_mat %<>% `colnames<-`(metadata_df$real_id) %>% `rownames<-`(metadata_df$real_id)

## vectorize again to identify whether relatedness pairs were consistent in the first place
relate_vec <- relate_mat %>% as.vector() %>% na.omit()
```

Coerce output to a vector

```r
# remove putative artefacts from clone info table
clone_allele_df <- clone_allele_df[1:771,]

# create empty matrix
```

```r
# row x col = samples x alleles
allele_mat <- matrix(nrow = length(unique(clone_allele_df$id)),
                     ncol = length(unique(clone_allele_df$allele))) %>%
  `rownames<-`(., as.character(unique(clone_allele_df$id))) %>%
  `colnames<-`(., as.character(
    str_sort(
      levels(
        clone_allele_df$allele)[1:19],
      numeric = T)))

# fill matrix with info on which and how many alleles are found
# in the clones for each individual fur seal
for (i in seq_along(unique(clone_allele_df$id))) {
  alleles_in_id <- summary(
    clone_allele_df$allele[clone_allele_df$id == unique(
      clone_allele_df$id)[i]])[1:19]

  allele_mat[i, ] <- alleles_in_id[str_sort(
    names(alleles_in_id),
    numeric = T)]
}

# otu_table handles taxa as rows. This case alleles are "taxa" in phylo tree
allele_mat <- ifelse(allele_mat != 0, 1, 0) %>%
  t()

# with artefacts, there are 30 allele levels.
# Make sure, to only keep putative alleles for analysis
allele_mat <- allele_mat[1:19,]


phyloseq_tree <- ape::read.tree("data/unifrac_tree_p.nwk")
# plot tree is interested
# plot(phyloseq_tree)

# create otu table for phyloseq object
arga_phylseq <- otu_table(allele_mat, taxa_are_rows = T)

# merge otu table and phyloseq tree
arga_phylseq <- merge_phyloseq(arga_phylseq, phyloseq_tree)

# create unifrac based on phyloseq
clone_gen.ufrac <- UniFrac(arga_phylseq, weighted = F) %>%
  # convert to distance matrix
  as.matrix()
```

UniFrac distance for MHC DQB II data

Plot genetic distance correlation Vectorize distance matrices based on microsatellite (msats_dist) and MHC (MHC_dist) genotypes:

```r
## Msats
## ---------------------------------------------------------------------------
# msats_gen.abs[upper.tri(msats_gen.abs, diag = T)] <- NA
```

```r
# msats_dist <- msats_gen.abs %>% as.vector() %>% na.omit()

## MHC
## ---------------------------------------------------------------------------
clone_gen.ufrac[upper.tri(clone_gen.ufrac)] <- NA
diag(clone_gen.ufrac) <- NA
MHC_dist <- clone_gen.ufrac %>% as.vector() %>% na.omit()

## combine in one data frame
## ---------------------------------------------------------------------------
df <- cbind(relate_vec, MHC_dist) %>% as.data.frame()
```

Generate linear model to test for correlation

```r
model <- lm(relate_vec ~ MHC_dist)
(m <- summary(model))
##
## Call:
## lm(formula = relate_vec ~ MHC_dist)
##
## Residuals:
##      Min       1Q   Median       3Q      Max
## -0.32583 -0.06280 -0.00340  0.05474  0.56823
##
## Coefficients:
##             Estimate Std. Error t value Pr(>|t|)
## (Intercept)  0.02776    0.01063   2.611  0.00912 **
## MHC_dist    -0.03637    0.01293  -2.812  0.00498 **
## ---
## Signif. codes:  0 '***' 0.001 '**' 0.01 '*' 0.05 '.' 0.1 ' ' 1
##
## Residual standard error: 0.1014 on 1538 degrees of freedom
## Multiple R-squared:  0.005116,   Adjusted R-squared:  0.004469
## F-statistic: 7.908 on 1 and 1538 DF,  p-value: 0.004984
```

Include individual IDs as a random factor

```r
create_pair_vars <-function(row_cross, col_cross, split_vars = F){
  require(stringr)

  rc <- row_cross
  cc <- col_cross

  # create empy matrix
  # keep row and col names from existing distance matrices

  empty_mat <- matrix(nrow = length(rc),
                      ncol = length(cc)) %>%
    `colnames<-`(cc) %>%
    `rownames<-`(rc)

  # fill each matrix i,j-th cell with the crossing from their corresponding
  # i-th rowname and j-th colname
  for (i in 1:dim(empty_mat)[1]) {
    for (j in 1:dim(empty_mat)[2]) {
```

```
      empty_mat[i,j] <- paste0(rc[i], "/", cc[j])

    } # end j
  } # end i


  # delete `upper.tri()` of `empty_mat` to resemble structure of the other
  # distance matrices in use

  empty_mat[upper.tri(empty_mat, diag = T)] <- NA
  pair_vars <- empty_mat %>% as.vector() %>% na.omit()

  # split `pair_vars` if needed
  if (split_vars == T) {

    pair_vars1 <- sapply(pair_vars,
                         function(x){
                           str_split(x, pattern = "/")[[1]][1]
                         })

    pair_vars2 <- sapply(pair_vars,
                         function(x){
                           str_split(x, pattern = "/")[[1]][2]
                         })

    pair_vars_split <- list(pair_variable1 = pair_vars1,
                            pair_variable2 = pair_vars2)

    return(pair_vars_split)

  } else {
    return(pair_vars)
  }

} #end create_pair_vars

# make vars

pairID1 <- create_pair_vars(row_cross = metadata_df$real_id,
                            col_cross = metadata_df$real_id,
                            split_vars = T)[1] %>%
  unlist() %>%
  as.vector()

pairID2 <- create_pair_vars(row_cross = metadata_df$real_id,
                            col_cross = metadata_df$real_id,
                            split_vars = T)[2] %>%
  unlist() %>%
  as.vector()
```

Create linear mixed effects model with pair IDs

```
rel_model_df <- data.frame(relate_vec, MHC_dist, pairID1, pairID2)
```

```r
m2 <- lmer(relate_vec ~ MHC_dist + (1|pairID1) + (1|pairID2), data = rel_model_df)
(store_m2 <- summary(model))
##
## Call:
## lm(formula = relate_vec ~ MHC_dist)
##
## Residuals:
##      Min       1Q   Median       3Q      Max
## -0.32583 -0.06280 -0.00340  0.05474  0.56823
##
## Coefficients:
##             Estimate Std. Error t value Pr(>|t|)
## (Intercept)  0.02776    0.01063   2.611  0.00912 **
## MHC_dist    -0.03637    0.01293  -2.812  0.00498 **
## ---
## Signif. codes:  0 '***' 0.001 '**' 0.01 '*' 0.05 '.' 0.1 ' ' 1
##
## Residual standard error: 0.1014 on 1538 degrees of freedom
## Multiple R-squared:  0.005116,	Adjusted R-squared:  0.004469
## F-statistic: 7.908 on 1 and 1538 DF,  p-value: 0.004984
```

Plot correlation of distance matrices

```r
figures[[3]] <- ggplot(df, aes(MHC_dist, relate_vec)) +
   geom_point() +
   # geom_jitter(height = 0.002, width = 0.07) +
   geom_smooth(method = "lm",
               formula = "y ~ x",
               se = T,
               color = "#fde725ff",
               fill = "#29af7fff",
               alpha = 0.38,
               size = 1.5) +
   scale_y_continuous(name   = "Relatedness") +
   scale_x_continuous(name   = "MHC UniFrac distance") +
   # annotate(geom = "text", x = 0.05, y = 69,
   #          label = "italic(R²)==0.01", parse = T) +
   theme_classic() +
   theme(
     panel.grid.minor = element_blank(),
     panel.grid.major = element_blank(),
     plot.margin = unit(c(0.5,1.5,0.5,0,5), "cm"),
     axis.title = element_text(color = "black",
                               margin = margin(10,10,10,10)),
     axis.text = element_text(color = "black")
   )
names(figures)[3] <- "relate_plot"
```

correlate Microsatellite and MHC DQB II heterozygosity

Calculate standardized multilocus heterozygosity in `inbreedR`

```r
## read msats data
## ---------------------------------------------------------------------------
df <- read.table("data/msats/msats_genotypes_inbreedR.txt", sep = "\t") %>%
```

```r
  # convert to inbreedR format
  convert_raw()

## check formatting
## ----------------------------------------------------------------------------
check_data(df)
## [1] TRUE
## Compute standardized multilocus heterozygosity
## ----------------------------------------------------------------------------
sMLH_res <- sMLH(df)

# histogram for interested
# hist(sMLH_res)
```

Convert mhc data into categorical hom/het values

```r
## Load MHC data
## ----------------------------------------------------------------------------
clones_het <- read.table(file = "data/clone_mhc_het.txt", sep = "\t")

## make rownames consistent
## ----------------------------------------------------------------------------
n <- names(sMLH_res)
n_c <- rownames(clones_het)
n_in <- match(n, n_c)
clones_het <- clones_het[n_in,]
```

Create data frame to ease modelling and plotting

```r
corr_het <- cbind(sMLH_res, clones_het$het) %>%
  `colnames<-`(c("smlh","mhc_het")) %>%
  as.data.frame()
```

Create glm

```r
## glm with binomially distributed data
## ----------------------------------------------------------------------------
het_glm <- glm(cbind(corr_het$mhc_het, 1-corr_het$mhc_het) ~ corr_het$smlh,
               family = "binomial")

summary(het_glm)
##
## Call:
## glm(formula = cbind(corr_het$mhc_het, 1 - corr_het$mhc_het) ~
##     corr_het$smlh, family = "binomial")
##
## Deviance Residuals:
##     Min       1Q   Median       3Q      Max
## -2.0457   0.4649   0.6023   0.7604   1.0006
##
## Coefficients:
##               Estimate Std. Error z value Pr(>|z|)
## (Intercept)      6.626      3.940   1.682   0.0927 .
## corr_het$smlh   -5.264      3.836  -1.372   0.1700
## ---
## Signif. codes:  0 '***' 0.001 '**' 0.01 '*' 0.05 '.' 0.1 ' ' 1
```

```
##
## (Dispersion parameter for binomial family taken to be 1)
##
##     Null deviance: 58.193  on 55  degrees of freedom
## Residual deviance: 56.172  on 54  degrees of freedom
## AIC: 60.172
##
## Number of Fisher Scoring iterations: 4
anova(het_glm, test = "Chisq") # test glm; chi square due to binomial data
## Analysis of Deviance Table
##
## Model: binomial, link: logit
##
## Response: cbind(corr_het$mhc_het, 1 - corr_het$mhc_het)
##
## Terms added sequentially (first to last)
##
##
##                Df Deviance Resid. Df Resid. Dev Pr(>Chi)
## NULL                              55     58.193
## corr_het$smlh  1   2.0212        54     56.172   0.1551
chi_glm <- qchisq(1 - 0.1551, df = 54, lower.tail = T)
```

Plot heterozygosity correlation (smlh on mhc)

```
figures[[4]] <- ggplot(data = corr_het,
                      aes(y = smlh,
                          x = mhc_het)) +
  geom_jitter(height = 0.02,
              width = 0.07,
              size = 2.5) +
  geom_smooth(method = "glm",
              formula = "y ~ x",
              color = "#fde725ff",
              fill = "#29af7fff",
              alpha = 0.38) +
  ylab("sMLH") +
  scale_x_continuous(name = "MHC heterozygosity",
                     breaks = c(0,1),
                     labels = c("homozygote", "heterozygote")) +
  theme_classic() + # base_size = 13,
                  # base_line_size = 1,
                  # base_rect_size = 1
  theme(
    # panel.background = element_rect(color = "black", size = 1.5),
    panel.grid = element_blank(),
    axis.text = element_text(colour = "black")
  )

names(figures)[4] <- "het_corr_plot"
```

Multipanel figure of MHC heterozygosity and diversity correlation

```
mhc_het.p <- figures[[4]] + labs(tag = "A") + theme(aspect.ratio = 0.7)
mhc_div.p <- figures[[3]] + labs(tag = "B") + theme(aspect.ratio = 0.7)

(ggpubr::ggarrange(mhc_het.p, mhc_div.p, nrow = 2, ncol = 1, align = "hv"))
```

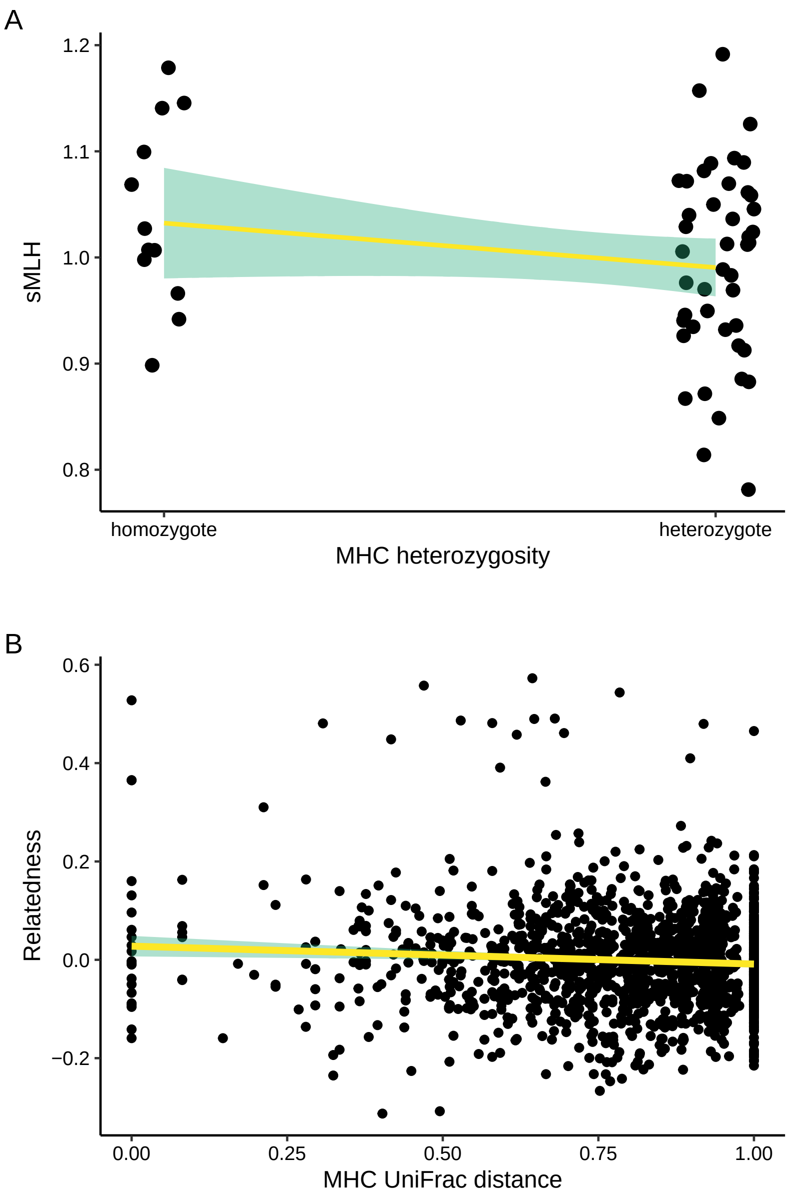


```
ggsave("graphics/mhc_het_div_panel.png", dpi = 400, width = 10, height = 7)
       # , width = 9, height = 7)
```

Allele detection curves and Hamming mismatches

Define functions

Calculate Hamming distances, simulate allele detection

```
## calculate pairwise difference to primer sequences.
## Optional, account for variable alignment length
## ---------------------------------------------------------------------------
Hamming.dist <- function(seq, ref, method = c("rel", "abs")) {
  method <- match.arg(method)
  # discard gaps and binding N
  gaps_seq <- which(seq %in% c("-", "N"))
  gaps_ref <- which(ref %in% c("-", "N"))
  gaps <- unique(c(gaps_seq, gaps_ref))

  seqx <- seq[-gaps]
  refx <- ref[-gaps]

  # estimate diff
  diff <- 0
  for (i in 1:length(seqx))  diff <- diff + ifelse(seqx[i] == refx[i], 0, 1)
  # correct for sequence length
  if (method == "rel") {
    diff <-
      ifelse(length(diff) > 0,diff/length(seqx), NA)
  }
  return(diff)
}# end Hamming.dist

## Pick alleles based on hamming value threshold
## ---------------------------------------------------------------------------
simulate_hoelzel <- function(data, n = 1:length(data), bs = 999,
                             hamming = hamming_values, mismatch = 1) {

  hamming <- subset(hamming, x <= mismatch)
  x <- rep(n, each = bs)
  y <- lapply(x, function(temp) {
    # sample genotypes
    get <- data[sample(x = 1:length(data),
                       size = temp,
                       replace = T)] %>%
      unlist() %>%
      unique()
    # keep alleles with < mismatch differences
    keep <- get[get %in% rownames(hamming)] %>%
      length()
  })

  df <- data.frame(x = x,y = unlist(y))
  df$x <- as.factor(df$x)
  return(df)
}# end simulate_hoelzel

#' @description Summarizes data
```

```r
#' @param data a data frame
#' @param measurevar character giving column name of data to summarise
#' @param groupvars character giving column names of grouping variables
#' @param na.rm boolean
#' @param conf.interval confidence interval (default 0.95)
#' @param .drop boolean
#'
#' @source
#' Taken from the R cookbook (cookbook-r.com/Manipulating_data/Summarizing_data/)
#'
summary_stats <- function(data = NULL,
                          measurevar = NULL,
                          groupvars = NULL,
                          na.rm = TRUE,
                          conf.interval = 0.95,
                          .drop = TRUE) {

  length2 <- function(x, na.rm = FALSE) {
    if (na.rm) {
      sum(!is.na(x))
    } else {
      length(x)
    }
  }
  # This does the summary. For each group's data frame, return a vector with
  # N, mean, and sd
  datac <- plyr::ddply(data, groupvars, .drop = .drop,
                       .fun = function(xx, col) {
                         c(N = length2(xx[[col]], na.rm = na.rm),
                           mean = mean(xx[[col]], na.rm = na.rm),
                           sd = sd(xx[[col]], na.rm = na.rm)
                         )
                       },
                       measurevar
  )

  # Rename the "mean" column
  datac <- plyr::rename(datac, c("mean" = measurevar))

  datac$se <- datac$sd / sqrt(datac$N)  # Calculate standard error of the mean

  # Confidence interval multiplier for standard error
  # Calculate t-statistic for confidence interval:
  # e.g., if conf.interval is .95, use .975 (above/below), and use df=N-1
  ciMult <- qt(conf.interval/2 + .5, datac$N - 1)
  datac$ci <- datac$se * ciMult

  return(datac)
}
```

Generate data for nucleotide mistmaches at PBR

```r
## Putative alleles Cloning sequences (full exon, 267bp)
## ---------------------------------------------------------------------------
Clones <- ape::read.dna("data/ArGa_DQB-Hoelzel-primer-clones_20211027.fas",
                         format = "fasta") %>%
  as.character() %>%
  apply(.,2, toupper) %>% ## append a dummy column
  cbind(., "-")
# ----------------------------------------------------------------------------

## Extract and remove primer from the alignment
ClonesPrimer <- Clones[1,]

## remove primer from matrix
Clones <- Clones[-1,]

Clones_hd <- data.frame(x = apply(Clones, 1,
                                  Hamming.dist,
                                  ref = ClonesPrimer,
                                  method = "abs") %>%
                          unlist())

# ----------------------------------------------------------------------------
Clones_glm_df <- data.frame(mismatches = Clones_hd$x,
                           binom = 0,
                           a_counts = c(145, 72, 62, 60, 57, 55, 54, 52, 48,
                                        46, 18, 17, 17, 14, 13, 13, 12, 9, 7))
## Set alleles characterised in Hoelzel et al to 1
Clones_glm_df$binom[c(6, 17)] <- 1
```

Plot mismatches

```r
set.seed(98)
figures[[5]] <- ggplot(Clones_glm_df,
                       aes(x = as.factor(binom),
                           y = mismatches,
                           fill = as.factor(binom))) +
    geom_boxplot(alpha = 0.9,
                 fatten = 3,
                 outlier.shape = NA ) +
    geom_jitter(aes(size = a_counts),
                shape = 21,
                alpha = 0.9,
                width = 0.4,
                height = 0.05,
                color = "black",
                fill = "grey") +
    scale_size(range = c(3,7)) +
    theme_classic(base_size = 16,
                  base_line_size = 1,
                  base_rect_size = 1) +
    scale_x_discrete(name = "Allele detected in both studies",
                     labels = c("No", "Yes")) +
    ylab("Mismatches at primer binding site") +
```

```
    labs(tag = "A") +
    scale_fill_manual(values = c("#FDE725FF", "#481567FF")) +
    theme(axis.ticks = element_line(color = "black"),
          axis.line = element_line(color = "black"),
          axis.text = element_text(color = "black"),
          legend.position = "none")

names(figures)[5] <- "hamming_boxplot"
```

Allele detection on simulated primer-mismatches

```
## Load genotypes
## -----------------------------------------------------------------------------
load("data/called_clones-20211027.RData")
clone_genotypes <- called_clones

## Simulate datasets
## -----------------------------------------------------------------------------
clone_simul <- lapply(0:max(Clones_hd), function(x) {
  simulate_hoelzel(data = clone_genotypes[["clone_exon"]],
                   bs = 99,
                   hamming = Clones_hd,
                   mismatch = x)
})

for (i in 1:length(clone_simul)) {
  clone_simul[[i]]$mismatches <- as.character(i - 1)
}

clone_simul <- do.call("rbind", clone_simul)
clone_simul$x <- as.numeric(as.character(clone_simul$x))
clone_summary <- summary_stats(clone_simul,
                               measurevar = "y",
                               groupvars = c("x","mismatches"),
                               conf.interval = 0.99)
## add number of Hoelzel et al., 1999
clone_summary[nrow(clone_summary) + 1, ] <- c(13, 99, 0, 4, 0, 0, 0)
clone_summary$x <- as.numeric(as.character(clone_summary$x))
```

Allele detection curves

Plot size does not match size of publication figure

```
hoelzel.exp <- c(expression("Hoelzel " * italic("et al.")))
figures[[6]] <- ggplot(clone_summary, aes(x,y)) +
  geom_linerange(ymin = clone_summary$y - clone_summary$sd,
                 ymax = clone_summary$y + clone_summary$sd,
                 col = "grey0",
                 alpha = 0.4) +
  geom_point(aes(shape = mismatches),
             size = 4,
             fill = "black") +
  xlab("Sample size") +
  ylab("Number of alleles detected") +
```

```r
  scale_x_continuous(breaks = seq(0,60,5)) +
  scale_y_continuous(breaks = seq(0,20,5),
                     limits = c(0,22)) +
  labs(tag = "B") +
  scale_shape_manual(labels = c("0 bp", "1 bp", "2 bp",
                                "3 bp", "4 bp", "5 bp",
                                hoelzel.exp),
                     breaks = c(0, 1, 2, 3, 4, 5, 99),
                     values = c(15, 0, 17, 2, 16, 1, 8)) +
  theme_classic(base_size = 16,
                base_line_size = 1,
                base_rect_size = 1) +
  theme(axis.ticks = element_line(color = "black"),
        axis.line = element_line(color = "black"),
        axis.text = element_text(color = "black"),
        legend.title = element_blank(),
        legend.background = element_rect(linetype = 1,
                                         color = "black"),
        legend.position = c(.0,1.0),
        legend.box.margin = margin(-5,0,0,8, "pt"),
        legend.justification = c("left", "top")) +
  guides(shape = guide_legend(ncol = 3,
                              label.hjust = 0))

addSmallLegend <- function(myPlot, pointSize = 0.5, textSize = 3, spaceLegend = 0.1) {
  myPlot +
    guides(shape = guide_legend(override.aes = list(size = pointSize),
                                ncol = 3,
                                label.hjust = 0),
           color = guide_legend(override.aes = list(size = pointSize))) +
    theme(legend.title = element_blank(),
          legend.text  = element_text(size = textSize),
          legend.key.size = unit(spaceLegend, "lines"))
}

figures[[6]] <- addSmallLegend(figures[[6]], pointSize = 3, textSize = 14)

names(figures)[6] <- "allele_detection_curve"
```

Plot as multi-panel figure

```r
ggpubr::ggarrange(figures[[5]], figures[[6]], nrow = 2, ncol = 1, align = "v")
```

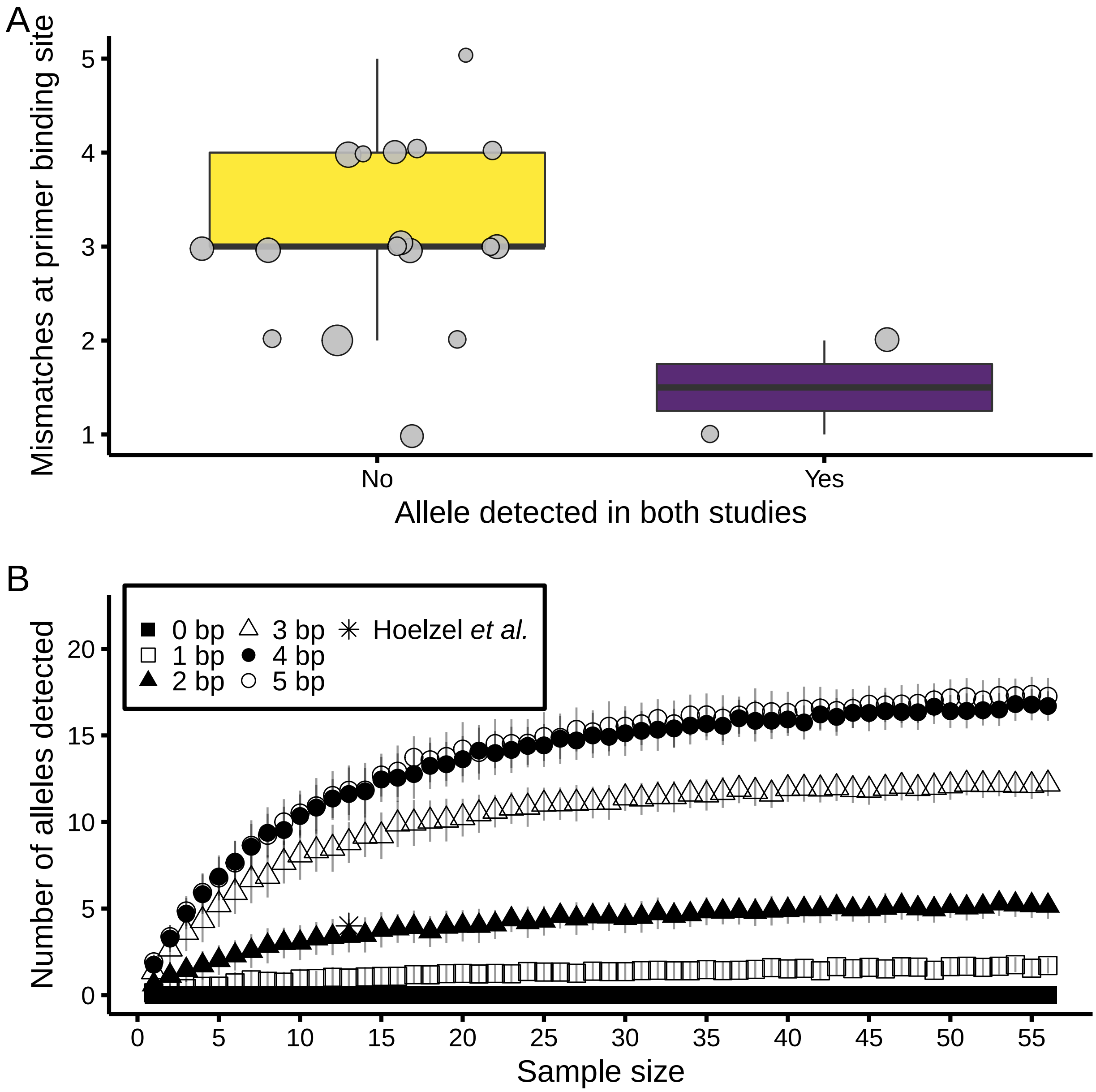

Constructing a phylogeny

```
# load newick file with unrooted tree
tree <- ape::read.tree("data/phylogeny/tree.nwk")
# root tree with `ape`
tree <- root(tree, node = 130, edgelabel = F, resolve.root = T)
# check root
is.rooted(tree)
## [1] TRUE
```

Create tip label and group information for plotting metadata

```
# get full names
tip_name <- tree$tip.label
tip_species <- sapply(
  tip_name,
  function(xx){
    z <- strsplit(xx, "_")[[1]][2]
    strsplit(z, "-")[[1]][1]
  }
) %>% unname()

# split genbank accessions
tip_acc <- sapply(
  tip_name,
  function(xx)
    strsplit(xx, "_")[[1]][1]
) %>% unname()

# combine information separate into data frame
tip_df <- paste0(tip_acc, " ", tip_species)
tip_df <- data.frame(label = tip_df,
                     accession = tip_acc,
                     species = tip_species) %>%
  `rownames<-`(NULL)

# factorize data
tip_df %<>% mutate(species = as.factor(species))

# Seemingly: for `%<+%` to work and join information of tip label with
# metadata, the first column of the assigned metadata frame and the tree$tip.label
# need to be same. Thus, we assign the new labels first and only afterwards join
# the metadata with the tree `Formal class treedata`
tree$tip.label <- tip_df$label

# dataframe needs update, too! Drop unused names and correct entries
tip_drop <- tip_df$label[63:67]
```

Collapse root tips to a master branch

```
tree <- drop.tip(tree, tip_drop)
tree$tip.label[63] <- "Canis lupus familiaris"
tip_df <- tip_df[1:63,]
tip_df[63,] <- c("Canis lupus familiaris", "AF016904-AF016909", "CaFa")
```

Plot phylogeny

```
species_names <- c("Arctocephalus forsteri",
                   "Arctocephalus gazella",
                   "Canis lupus familiaris",
                   "Halichoerus grypus",
                   "Mirounga angustirostris",
                   "Mirounga leonina",
```

```r
                   "Monachus schauinslandi",
                   "Neophoca cinerea",
                   "Odobenus rosmarus",
                   "Phocarctos hookeri",
                   "Zalophus californianus",
                   "Zalophus wollebaeki")

species_palette <- RColorBrewer::brewer.pal(12, "Set3")
species_palette[c(2,3, 12)] <- c("black", "#a76437","red")

# create initial tree
p <- ggtree(tree,
            layout = "circular",
            ladderize = F)
# provide metadata: `%<+%`
p <- p %<+% tip_df +
  geom_tiplab2(size = 5,
               align = F,
               hjust = -0.05) +
  geom_rootedge(rootedge = 0.05) +
  scale_color_manual(values = species_palette,
                     labels = species_names) +
  geom_tippoint(aes(color = species),
                size = 3) +
  geom_treescale(x = 0,
                 linesize = 1.3,
                 offset = 1) +
  xlim_tree(.35) +
  labs(color = "Species") +
  theme(
    legend.position = "right"
  )
p + guides(color = guide_legend(title.position = "top"))
```

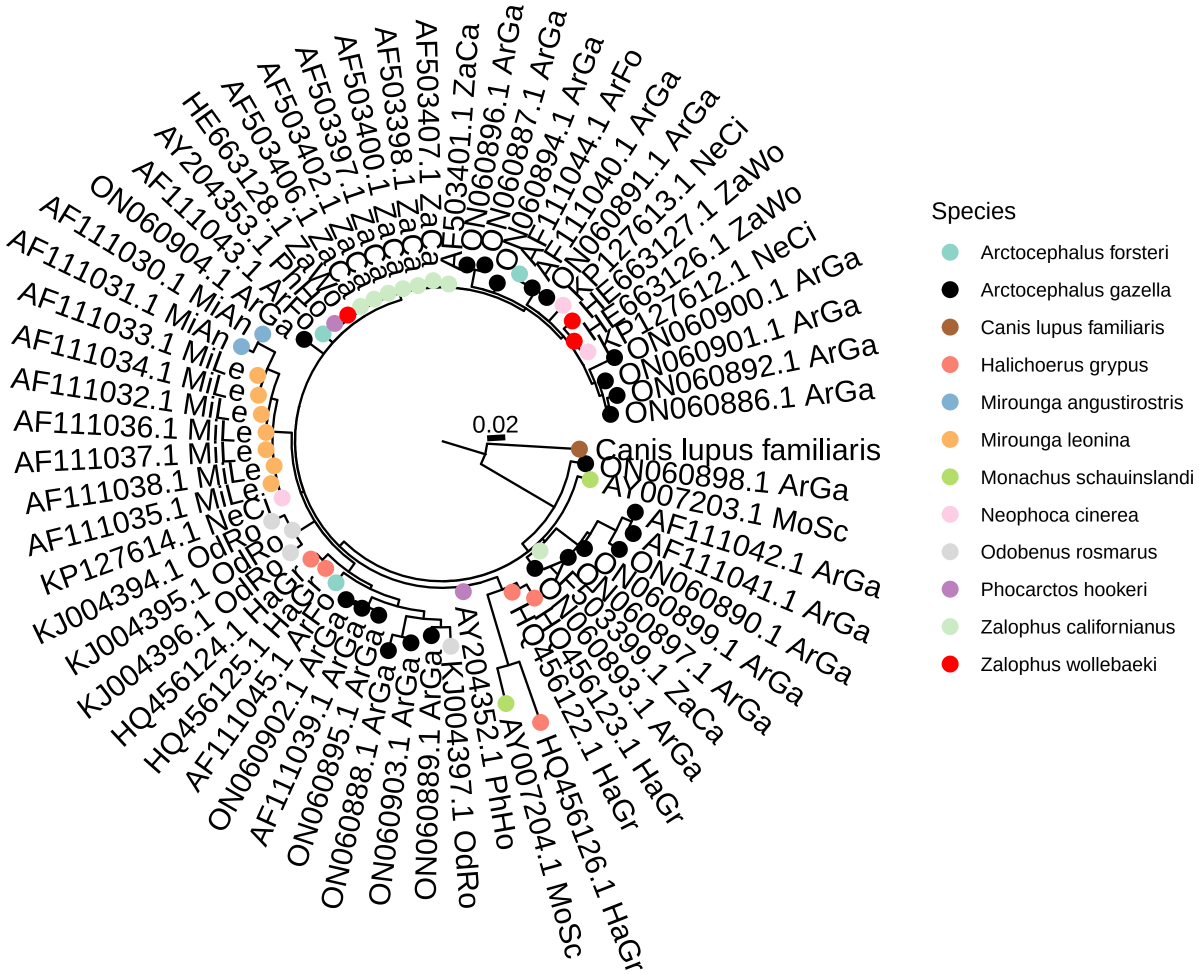


```r
addSmallLegend <- function(myPlot, pointSize = 0.5, textSize = 3, spaceLegend = 0.1) {
  myPlot +
    guides(shape = guide_legend(override.aes = list(size = pointSize),
                                ncol = 3,
                                label.hjust = 0),
           color = guide_legend(override.aes = list(size = pointSize))) +
    theme(legend.title = element_blank(),
          legend.text  = element_text(size = textSize),
          legend.key.size = unit(spaceLegend, "lines"))
}

p <- addSmallLegend(p, pointSize = 3, textSize = 14)

# ggsave("graphics/phyl_new.png", dpi = 400, width = 50.24, height = 32.88, units = "cm")
```

Session information

```
sessionInfo()
## R version 4.2.1 (2022-06-23 ucrt)
## Platform: x86_64-w64-mingw32/x64 (64-bit)
## Running under: Windows 10 x64 (build 19044)
##
## Matrix products: default
##
## locale:
## [1] LC_COLLATE=German_Germany.utf8  LC_CTYPE=German_Germany.utf8
## [3] LC_MONETARY=German_Germany.utf8 LC_NUMERIC=C
## [5] LC_TIME=German_Germany.utf8
##
## attached base packages:
## [1] stats4    stats     graphics  grDevices utils     datasets  methods
## [8] base
##
## other attached packages:
##  [1] ggtree_3.4.1        treeio_1.20.1       egg_0.4.5
##  [4] ggpubr_0.4.0        gridExtra_2.3       magrittr_2.0.3
##  [7] RColorBrewer_1.1-3  reshape2_1.4.4      patchwork_1.1.1
## [10] forcats_0.5.1       stringr_1.4.0       dplyr_1.0.9
## [13] purrr_0.3.4         readr_2.1.2         tidyr_1.2.0
## [16] tibble_3.1.7        ggplot2_3.3.6       tidyverse_1.3.2
## [19] vegan_2.6-2         lattice_0.20-45     permute_0.9-7
## [22] poppr_2.9.3         phyloseq_1.40.0     lme4_1.1-30
## [25] Matrix_1.4-1        inbreedR_0.3.3      hierfstat_0.5-11
## [28] genepop_1.1.7       EnvStats_2.7.0      Demerelate_0.9-3
## [31] Biostrings_2.64.0   GenomeInfoDb_1.32.2 XVector_0.36.0
## [34] IRanges_2.30.0      S4Vectors_0.34.0    BiocGenerics_0.42.0
## [37] BiocManager_1.30.18 ape_5.6-2           adegenet_2.1.7
## [40] ade4_1.7-19
##
## loaded via a namespace (and not attached):
##   [1] readxl_1.4.0           backports_1.4.1        plyr_1.8.7
##   [4] igraph_1.3.4           lazyeval_0.2.2         fts_0.9.9.2
##   [7] splines_4.2.1          digest_0.6.29          yulab.utils_0.0.5
##  [10] foreach_1.5.2          htmltools_0.5.3        fansi_1.0.3
##  [13] googlesheets4_1.0.0    cluster_2.1.3          sfsmisc_1.1-13
##  [16] tzdb_0.3.0             modelr_0.1.8           colorspace_2.0-3
##  [19] rvest_1.0.2            mlogit_1.1-1           haven_2.5.0
##  [22] rbibutils_2.2.8        xfun_0.31              crayon_1.5.1
##  [25] RCurl_1.98-1.7         jsonlite_1.8.0         survival_3.3-1
##  [28] zoo_1.8-10             iterators_1.0.14       glue_1.6.2
##  [31] gtable_0.3.0           gargle_1.2.0           zlibbioc_1.42.0
##  [34] seqinr_4.2-16          polysat_1.7-6          car_3.1-0
##  [37] Rhdf5lib_1.18.2        abind_1.4-5            scales_1.2.0
##  [40] DBI_1.1.3              rstatix_0.7.0          Rcpp_1.0.9
##  [43] viridisLite_0.4.0      xtable_1.8-4           gridGraphics_0.5-1
##  [46] tidytree_0.3.9         Formula_1.2-4          httr_1.4.3
##  [49] ellipsis_0.3.2         farver_2.1.1           pkgconfig_2.0.3
##  [52] dbplyr_2.2.1           utf8_1.2.2             labeling_0.4.2
##  [55] ggplotify_0.1.0        tidyselect_1.1.2       rlang_1.0.4
```

```
##  [58] later_1.3.0           munsell_0.5.0       cellranger_1.1.0
##  [61] tools_4.2.1           cli_3.3.0           generics_0.1.3
##  [64] broom_1.0.0           evaluate_0.15       biomformat_1.24.0
##  [67] fastmap_1.1.0         yaml_2.3.5          knitr_1.39
##  [70] fs_1.5.2              nlme_3.1-157        mime_0.12
##  [73] aplot_0.1.6           dfidx_0.0-5         xml2_1.3.3
##  [76] compiler_4.2.1        rstudioapi_0.13     ggsignif_0.6.3
##  [79] reprex_2.0.1          statmod_1.4.36      stringi_1.7.8
##  [82] nloptr_2.0.3          multtest_2.52.0     vctrs_0.4.1
##  [85] pillar_1.8.0          lifecycle_1.0.1     rhdf5filters_1.8.0
##  [88] Rdpack_2.3.1          lmtest_0.9-40       cowplot_1.1.1
##  [91] data.table_1.14.2     bitops_1.0-7        httpuv_1.6.5
##  [94] R6_2.5.1              promises_1.2.0.1    codetools_0.2-18
##  [97] boot_1.3-28           MASS_7.3-57         assertthat_0.2.1
## [100] rhdf5_2.40.0          withr_2.5.0         GenomeInfoDbData_1.2.8
## [103] pegas_1.1             mgcv_1.8-40         parallel_4.2.1
## [106] hms_1.1.1             ggfun_0.0.6         grid_4.2.1
## [109] minqa_1.2.4           rmarkdown_2.14      carData_3.0-5
## [112] googledrive_2.0.0     Biobase_2.56.0      shiny_1.7.2
## [115] lubridate_1.8.0
```

References

Arora, Sonali, Martin Morgan, Marc Carlson, and H. Pagès. 2021. *GenomeInfoDb: Utilities for Manipulating Chromosome Names, Including Modifying Them to Follow a Particular Naming Style.* https://bioconductor.org/packages/GenomeInfoDb.

Auguie, Baptiste. 2017. *gridExtra: Miscellaneous Functions for "Grid" Graphics.* https://CRAN.R-project.org/package=gridExtra.

———. 2019. *Egg: Extensions for Ggplot2: Custom Geom, Custom Themes, Plot Alignment, Labelled Panels, Symmetric Scales, and Fixed Panel Size.* https://CRAN.R-project.org/package=egg.

Bache, Stefan Milton, and Hadley Wickham. 2022. *Magrittr: A Forward-Pipe Operator for r.* https://CRAN.R-project.org/package=magrittr.

Bougeard, Stéphanie, and Stéphane Dray. 2018. "Supervised Multiblock Analysis in R with the ade4 Package." *Journal of Statistical Software* 86 (1): 1–17. https://doi.org/10.18637/jss.v086.i01.

Chessel, Daniel, Anne-Béatrice Dufour, and Jean Thioulouse. 2004. "The ade4 Package – I: One-Table Methods." *R News* 4 (1): 5–10. https://cran.r-project.org/doc/Rnews/.

Dray, StÃ©phane, Anne-BÃ©atrice Dufour, Jean Thioulouse, Thibaut Jombart, Sandrine Pavoine, Jean R. Lobry, SÃ©bastien Ollier, AurÃ©lie Siberchicot, and Daniel Chessel. 2021. *Ade4: Analysis of Ecological Data: Exploratory and Euclidean Methods in Environmental Sciences.* http://pbil.univ-lyon1.fr/ADE-4/.

Dray, Stéphane, and Anne–Béatrice Dufour. 2007. "The ade4 Package: Implementing the Duality Diagram for Ecologists." *Journal of Statistical Software* 22 (4): 1–20. https://doi.org/10.18637/jss.v022.i04.

Dray, Stéphane, Anne-Béatrice Dufour, and Daniel Chessel. 2007. "The ade4 Package – II: Two-Table and K-Table Methods." *R News* 7 (2): 47–52. https://cran.r-project.org/doc/Rnews/.

Goudet, Jerome, and Thibaut Jombart. 2021. *Hierfstat: Estimation and Tests of Hierarchical f-Statistics.* https://CRAN.R-project.org/package=hierfstat.

Henry, Lionel, and Hadley Wickham. 2020. *Purrr: Functional Programming Tools.* https://CRAN.R-project.org/package=purrr.

Huber, W., Carey, V. J., Gentleman, R., Anders, et al. 2015. "Orchestrating High-Throughput Genomic Analysis with Bioconductor." *Nature Methods* 12 (2): 115–21. http://www.nature.com/nmeth/journal/v12/n2/full/nmeth.3252.html.

Jombart, T. 2008. "Adegenet: A r Package for the Multivariate Analysis of Genetic Markers." *Bioinformatics* 24: 1403–5. https://doi.org/10.1093/bioinformatics/btn129.

Jombart, T., and I. Ahmed. 2011. "Adegenet 1.3-1: New Tools for the Analysis of Genome-Wide SNP Data." *Bioinformatics.* https://doi.org/10.1093/bioinformatics/btr521.

Jombart, Thibaut, and Zhian N. Kamvar. 2021. *Adegenet: Exploratory Analysis of Genetic and Genomic Data*. https://github.com/thibautjombart/adegenet.

Kamvar, Zhian N., Jonah C. Brooks, and Niklaus J. Grünwald. 2015. "Novel R tools for analysis of genome-wide population genetic data with emphasis on clonality." *Front. Genet.* 6 (June): 208. https://doi.org/10.3389/fgene.2015.00208.

Kamvar, Zhian N., Javier F. Tabima, Jonah C. Brooks, and David Folarin. 2021. *Poppr: Genetic Analysis of Populations with Mixed Reproduction*. https://CRAN.R-project.org/package=poppr.

Kamvar, Zhian N., Javier F. Tabima, and Niklaus J. Grünwald. 2014. "*Poppr*: An R Package for Genetic Analysis of Populations with Clonal, Partially Clonal, and/or Sexual Reproduction." *PeerJ* 2 (March): e281. https://doi.org/10.7717/peerj.281.

Kassambara, Alboukadel. 2020. *Ggpubr: Ggplot2 Based Publication Ready Plots*. https://rpkgs.datanovia.com/ggpubr/.

Lawrence, Michael, Wolfgang Huber, Hervé Pagès, Patrick Aboyoun, Marc Carlson, Robert Gentleman, Martin Morgan, and Vincent Carey. 2013. "Software for Computing and Annotating Genomic Ranges." *PLoS Computational Biology* 9. https://doi.org/10.1371/journal.pcbi.1003118.

McMurdie, Paul J., and Susan Holmes. 2013. "Phyloseq: An r Package for Reproducible Interactive Analysis and Graphics of Microbiome Census Data." *PLoS ONE* 8 (4): e61217. http://dx.plos.org/10.1371/journal.pone.0061217.

McMurdie, Paul J., Susan Holmes, with contributions from Gregory Jordan, and Scott Chamberlain. 2021. *Phyloseq: Handling and Analysis of High-Throughput Microbiome Census Data*. http://dx.plos.org/10.1371/journal.pone.0061217.

Millard, Steven P. 2013. *EnvStats: An r Package for Environmental Statistics*. New York: Springer. https://www.springer.com.

———. 2022. *EnvStats: Package for Environmental Statistics, Including US EPA Guidance*. https://github.com/alexkowa/EnvStats.

Müller, Kirill, and Hadley Wickham. 2021. *Tibble: Simple Data Frames*. https://CRAN.R-project.org/package=tibble.

Neuwirth, Erich. 2014. *RColorBrewer: ColorBrewer Palettes*. https://CRAN.R-project.org/package=RColorBrewer.

Oksanen, Jari, F. Guillaume Blanchet, Michael Friendly, Roeland Kindt, Pierre Legendre, Dan McGlinn, Peter R. Minchin, et al. 2020. *Vegan: Community Ecology Package*. https://CRAN.R-project.org/package=vegan.

Pagès, H., P. Aboyoun, R. Gentleman, and S. DebRoy. 2021. *Biostrings: Efficient Manipulation of Biological Strings*. https://bioconductor.org/packages/Biostrings.

Pagès, H., P. Aboyoun, and M. Lawrence. 2021. *IRanges: Foundation of Integer Range Manipulation in Bioconductor*. https://bioconductor.org/packages/IRanges.

Pagès, Hervé, and Patrick Aboyoun. 2021. *XVector: Foundation of External Vector Representation and Manipulation in Bioconductor*. https://bioconductor.org/packages/XVector.

Pagès, H., M. Lawrence, and P. Aboyoun. 2021. *S4Vectors: Foundation of Vector-Like and List-Like Containers in Bioconductor*. https://bioconductor.org/packages/S4Vectors.

Paradis, Emmanuel, Simon Blomberg, Ben Bolker, Joseph Brown, Santiago Claramunt, Julien Claude, Hoa Sien Cuong, et al. 2022. *Ape: Analyses of Phylogenetics and Evolution*. http://ape-package.ird.fr/.

Paradis, E., and K. Schliep. 2019. "Ape 5.0: An Environment for Modern Phylogenetics and Evolutionary Analyses in R." *Bioinformatics* 35: 526–28.

Pedersen, Thomas Lin. 2020. *Patchwork: The Composer of Plots*. https://CRAN.R-project.org/package=patchwork.

R Core Team. 2021. *R: A Language and Environment for Statistical Computing*. Vienna, Austria: R Foundation for Statistical Computing. https://www.R-project.org/.

Rousset, François. 2008. "Genepop'007: A Complete Re-Implementation of the Genepop Software for Windows and Linux." *Molecular Ecology Resources* 8: 103–6.

———. 2020. *Genepop: Population Genetic Data Analysis Using Genepop*. https://CRAN.R-project.org/package=genepop.

Sarkar, Deepayan. 2008. *Lattice: Multivariate Data Visualization with r*. New York: Springer. http://lmdvr.r-forge.r-project.org.

———. 2021. *Lattice: Trellis Graphics for r*. http://lattice.r-forge.r-project.org/.

Simpson, Gavin L. 2022. *Permute: Functions for Generating Restricted Permutations of Data*. https://github.com/gavinsimpson/permute.

Stoffel, Martin A., Mareike Esser, Joseph Hoffman, and Marty Kardos. 2022. *inbreedR: Analysing Inbreeding Based on Genetic Markers*. https://CRAN.R-project.org/package=inbreedR.

Stoffel, Martin A., Mareike Esser, Marty Kardos, Emily Humble, Hazel Nichols, Patrice David, and Joseph I. Hoffman. 2016. "inbreedR: An r Package for the Analysis of Inbreeding Based on Genetic Markers." *Methods in Ecology and Evolution*. https://doi.org/10.1111/2041-210X.12588.

Team, The Bioconductor Dev. 2021. *BiocGenerics: S4 Generic Functions Used in Bioconductor*. https://bioconductor.org/packages/BiocGenerics.

Thioulouse, Jean, Stéphane Dray, Anne–Béatrice Dufour, Aurélie Siberchicot, Thibaut Jombart, and Sandrine Pavoine. 2018. *Multivariate Analysis of Ecological Data with ade4*. Springer. https://doi.org/10.1007/978-1-4939-8850-1.

Wickham, Hadley. 2007. "Reshaping Data with the reshape Package." *Journal of Statistical Software* 21 (12): 1–20. http://www.jstatsoft.org/v21/i12/.

———. 2016. *Ggplot2: Elegant Graphics for Data Analysis*. Springer-Verlag New York. https://ggplot2.tidyverse.org.

———. 2019. *Stringr: Simple, Consistent Wrappers for Common String Operations*. https://CRAN.R-project.org/package=stringr.

———. 2020. *Reshape2: Flexibly Reshape Data: A Reboot of the Reshape Package*. https://github.com/hadley/reshape.

———. 2021a. *Forcats: Tools for Working with Categorical Variables (Factors)*. https://CRAN.R-project.org/package=forcats.

———. 2021b. *Tidyverse: Easily Install and Load the Tidyverse*. https://CRAN.R-project.org/package=tidyverse.

Wickham, Hadley, Mara Averick, Jennifer Bryan, Winston Chang, Lucy D'Agostino McGowan, Romain François, Garrett Grolemund, et al. 2019. "Welcome to the tidyverse." *Journal of Open Source Software* 4 (43): 1686. https://doi.org/10.21105/joss.01686.

Wickham, Hadley, Winston Chang, Lionel Henry, Thomas Lin Pedersen, Kohske Takahashi, Claus Wilke, Kara Woo, Hiroaki Yutani, and Dewey Dunnington. 2021. *Ggplot2: Create Elegant Data Visualisations Using the Grammar of Graphics*. https://CRAN.R-project.org/package=ggplot2.

Wickham, Hadley, Romain François, Lionel Henry, and Kirill Müller. 2022. *Dplyr: A Grammar of Data Manipulation*. https://CRAN.R-project.org/package=dplyr.

Wickham, Hadley, and Maximilian Girlich. 2022. *Tidyr: Tidy Messy Data*. https://CRAN.R-project.org/package=tidyr.

Wickham, Hadley, Jim Hester, and Jennifer Bryan. 2022. *Readr: Read Rectangular Text Data*. https://CRAN.R-project.org/package=readr.
